# Treatments for enhancing sleep quality in fibromyalgia: a systematic review and meta-analysis

**DOI:** 10.1093/rheumatology/keaf147

**Published:** 2025-03-14

**Authors:** Anna Pathak, Eoin M Kelleher, Isabelle Brennan, Raj Amarnani, Amanda Wall, Robert Murphy, Hopin Lee, Beth Fordham, Anushka Irani

**Affiliations:** Department of Medical Sciences, University of Oxford, Oxford, UK; Department of General Surgery, NHS Oxford University Hospitals Trust, Oxford, UK; Wellcome Centre for Integrative Neuroimaging, FMRIB Building, John Radcliffe Hospital, Oxford, UK; School of Clinical Medicine, University of Cambridge, Cambridge, UK; Rheumatology Department, University College London Hospitals NHS Foundation Trust, London, UK; Wellcome Centre for Integrative Neuroimaging, FMRIB Building, John Radcliffe Hospital, Oxford, UK; Department of Medical Sciences, University of Oxford, Oxford, UK; Oxford NIHR Musculoskeletal Biomedical Research Unit, Nuffield Department of Orthopaedics, Rheumatology and Musculoskeletal Sciences, University of Oxford, Oxford, UK; Oxford NIHR Musculoskeletal Biomedical Research Unit, Nuffield Department of Orthopaedics, Rheumatology and Musculoskeletal Sciences, University of Oxford, Oxford, UK; Oxford NIHR Musculoskeletal Biomedical Research Unit, Nuffield Department of Orthopaedics, Rheumatology and Musculoskeletal Sciences, University of Oxford, Oxford, UK; Division of Rheumatology, Mayo Clinic Florida, Jacksonville, FL, USA

**Keywords:** fibromyalgia, sleep, pharmacological therapies, cognitive behavioural therapy

## Abstract

**Objectives:**

Sleep disturbance is a key symptom of fibromyalgia and a risk factor for chronic widespread pain. This systematic review and meta-analysis aims to assess the effectiveness of pharmacological treatments and cognitive behavioural therapy (CBT) in improving sleep quality in fibromyalgia patients.

**Methods:**

A systematic search of PubMed, MEDLINE, Embase, Cochrane CENTRAL and CINAHL was conducted for randomized controlled trials (RCTs) published up to April 2023. Studies assessing pharmacological or CBT interventions with sleep-related outcomes were included. Data were extracted, and meta-analyses were performed where applicable. Study quality and bias were evaluated using the Cochrane Risk of Bias tool.

**Results:**

Forty-seven RCTs, including 11 094 participants, were reviewed. CBT for insomnia (CBT-I) showed a significant improvement in sleep quality (SMD −0.63, 95% CI −0.98 to −0.27), while CBT for pain (CBT-P) had no significant impact. Pharmacological agents such as pregabalin and sodium oxybate moderately improved sleep, but there was uncertainty around this evidence. Amitriptyline, milnacipran and duloxetine showed no significant benefit for sleep. Study heterogeneity was moderate, and no publication bias was detected.

**Conclusion:**

CBT-I is a promising treatment for enhancing sleep quality in fibromyalgia. Pharmacological treatments like pregabalin may be beneficial but should be used cautiously due to potential risks. Future research should prioritize trials focusing on sleep as a primary outcome and explore the comparative effectiveness of pharmacological treatments and CBT-I in fibromyalgia. Understanding the mechanisms linking sleep and fibromyalgia will also help guide future therapies.

## Graphical abstract

**Figure:**
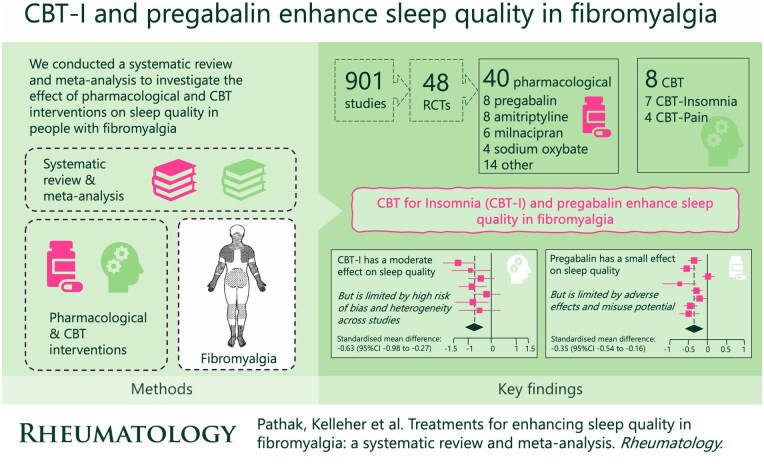

Rheumatology key messages

- CBT-I is a promising treatment for improving sleep in fibromyalgia.
- Pregabalin improves sleep but carries risks, limiting its use as a first-line therapy.
- Further trials should focus on sleep as a primary outcome in fibromyalgia treatment.

## Introduction

Fibromyalgia is a debilitating chronic pain condition characterized by widespread pain accompanied by sleep difficulties, cognitive impairment, mood disturbance and fatigue [1–3]. Sleep disturbance is central to fibromyalgia [4], and the James Lind Alliance Priority Setting Partnership emphasizes the importance of improving management of sleep disturbance in fibromyalgia [5]. However, current clinical guidance for the management of sleep disturbance in fibromyalgia lacks clear consensus [6–8]. The most recent EULAR guidance recommends meditative movement, amitriptyline, cyclobenzaprine and pregabalin to improve sleep in the context of fibromyalgia [6]. However, apart from amitriptyline, none of these therapies were recommended by the UK’s National Institute for Health and Care Excellence (NICE) 2021 guidelines for the management of chronic pain (NG193 [9]), and further research on cognitive behavioural therapy for insomnia (CBT-I) was encouraged.

CBT-based approaches have a substantial evidence base to support their effectiveness in improving quality of life, physical and mental health for people living with a range of physical and psychological health problems [10], but its effectiveness at improving sleep in fibromyalgia is less certain.

The aim of this systematic review is to identify and synthesize the current evidence on the effectiveness of pharmacological and CBT therapies in improving sleep quality in people with fibromyalgia.

## Methods

### Search strategy and selection criteria

A systematic review was conducted in accordance with the strategy recommended by the Cochrane Collaboration handbook [11]. Searches were conducted across PubMed, MEDLINE, Embase, Cochrane CENTRAL and CINAHL, using keywords related to pharmacological treatments or CBT in fibromyalgia (Supplementary Data S1, available at *Rheumatology* online). The search was performed to include studies published up to April 2023. Additional records were identified through a snowball search of references and clinical trial registries. Duplicates were removed, and the title and abstracts of remaining articles were reviewed by four researchers to identify studies suitable for inclusion based. The full texts of remaining articles were reviewed by the same researchers to determine their relevance. After this, four authors independently reviewed all resulting papers for eligibility. Discrepancies were resolved through discussion at each stage, and consensus was achieved.

*Inclusion criteria:*

1. Original research from randomized controlled trials (RCTs). Non-randomized trials and observational studies were excluded.
2. Published in English or translated to English.
3. Participants with a diagnosis of fibromyalgia based on the ACR 1990, 2010 or 2016 diagnostic criteria [12], the ACTTION-APS Pain Taxonomy (AAPT) diagnostic criteria (Arnold *et al.* [13]) or the German Association of the Scientific Medical Societies (AWMF) diagnostic criteria [14].
4. Pharmacological or CBT interventions with quantitative self-report or objective measures of sleep quality as an outcome. Studies that did not measure sleep quality directly were excluded.
5. For pharmacological therapies, the control intervention was required to be a placebo medication of the same appearance taken in the same regime.
6. For CBT studies, CBT had to be delivered by therapists trained in CBT or via the internet with an individual programme based on the principles of CBT. All modalities of CBT were included as defined by a recent health technology assessment [10]. Studies with combined interventions were excluded (e.g., physiotherapy with CBT).

### Study quality and risk of bias

Three authors assessed the risk of bias using the Cochrane Risk of Bias tool [15], evaluating the following domains: random sequence generation, allocation concealment, blinding of study participants, blinding of study personnel, blinding of outcome assessors, incomplete outcome data addressed, selective reporting and other potential bias. Each study was rated as low risk, some concerns or high risk. Studies with missing baseline differences, adverse events or incomplete data were considered high risk for reporting bias. The researchers independently evaluated all studies and resolved any discrepancies through discussion.

### Data extraction

Data on participants, interventions and results were extracted using Microsoft Forms. For studies with multiple interventions, data were recorded separately for each intervention group. Two researchers independently extracted the data for each study, resolving discrepancies through discussion. In the case of missing data, attempts were made to contact primary authors for further information.

### Meta analysis

A meta-analysis was conducted for interventions with sleep outcomes reported in more than three studies. Effect sizes were calculated as standardized mean differences (SMD), accounting for baseline differences between groups where possible. For parallel studies, the SMD was computed as the mean difference between the treatment and control groups, divided by the pooled standard deviation (SD). When available, baseline-adjusted change scores were used to determine SMD. The standard error (SE) for each SMD was computed based on the individual group SDs and sample sizes. As three of the four milnacipran studies reported mean differences at follow-up, these were converted into SMDs. For crossover studies, the pooled SD of differences was used, accounting for within-subject correlation (assumed at 0.5 if not otherwise reported). Studies with missing post-treatment outcome data were excluded, while missing variance data were imputed using baseline or the closest similar study. When studies included multiple treatment doses, the effects were pooled using inverse variance weighting, and subgroup analyses stratified by dosage group were also performed. In cases where multiple sleep measurement methods were used (e.g. multiple self-report items or multiple physiological measures), validated tools (e.g. Jenkins Sleep Scale [JSS], Medical Outcomes Study Sleep Scale [MOS-SS], Pittsburgh sleep quality index[PSQI]) were preferentially chosen, and the most consistently used methods within the selected study group were extracted to improve comparison across the studies. Pooled effect sizes were interpreted as small (SMD 0.2), moderate (SMD 0.5) and large (SMD 0.8). Results were considered statistically significant if *P* < 0.05.

A random-effects model was applied to account for variability between studies, with restricted maximum likelihood (REML) used to estimate tau-squared. The primary outcome measure was the pooled SMD with 95% CIs. Heterogeneity was quantified using the I^2^ statistic, with values >50% indicating substantial heterogeneity. Cochran’s Q-test (*P* < 0.1) was used to assess statistical significance of heterogeneity. Prediction intervals were reported to estimate the range of potential effects in future studies [16].

Publication bias was assessed through visual inspection of funnel plots and Egger’s regression test when ≥6 studies were available. Leave-one-out sensitivity analyses were conducted by removing each study in turn to evaluate the impact on the overall pooled effect. Influence analyses assessed the contribution of individual studies to heterogeneity and overall effect size.

Statistical analyses were conducted using R software (v4.4.1), using the ‘esc’, ‘meta’ and ‘metafor’ packages.

## Results

### Study selection

**Figure 1.**
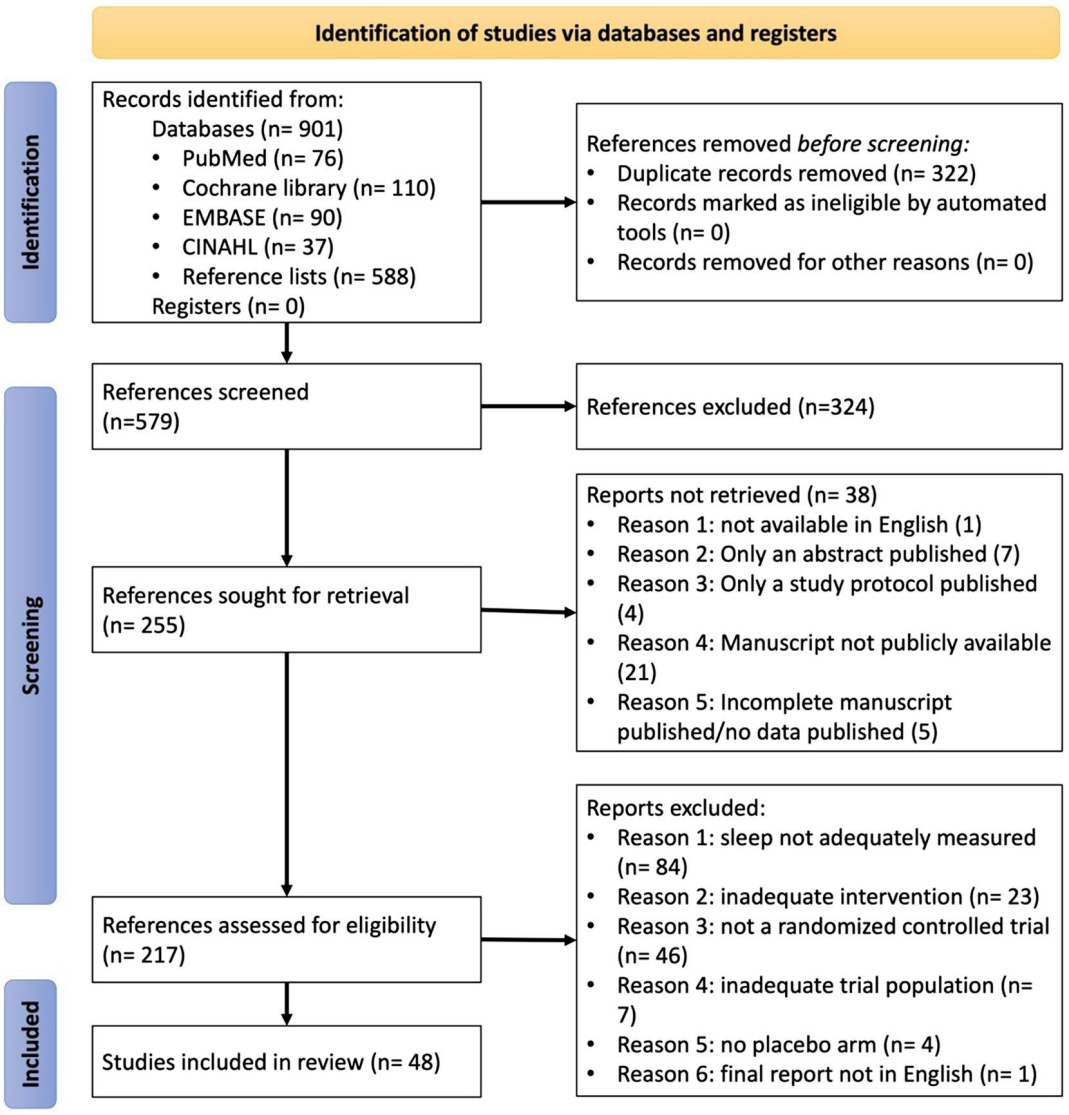
Flow diagram summarizing literature search presented according to PRISMA guidelines

The initial search yielded 901 studies, with 322 duplicates excluded (Fig. 1). After screening 579 titles and abstracts, 325 were removed, and the full texts of 255 articles were reviewed. Of these, 217 were assessed against inclusion criteria, resulting in 48 RCTs being included in the review [17–64].

### Study characteristics

This review included 11 140 (10 608 pharmacological; 532 CBT) participants with a mean (SD) age of 47.28 (4), of which 95.5% were female. Sample sizes varied widely, with a median (range) sample size of 125 (19–1196) participants for the pharmacological studies, and 64 (26–148) for the CBT studies. The median (range) duration of treatment was 12 weeks (4–27) for pharmacological studies and 8 weeks (6–14) for CBT. Recruitment was reported from hospital outpatients and the community, but the recruitment methodology was unclear in most studies. Clinical trial registration details are given in Supplementary Table S1, available at *Rheumatology* online.

Pharmacological therapies were evaluated in 40 studies [17–46, 52–60, 64] (Table 1), and CBT interventions were evaluated in eight studies [47–51, 61–63] (Table 2).

**Table 1.** Characteristics and outcomes of included pharmacological studies for sleep quality in patients with fibromyalgia

Drug	Study	Setting	Randomized, *N*	Age, years	% Female	Groups (dose)	Treatment duration, weeks	Sleep measure(s)	Follow-up, weeks	Completed study, %	Risk of bias	Change from baseline	Overall result	Notes
Acetyl-l-carnitine (LAC)	Rossini *et al.* 2007 [52]	7 sites in Italy	102	LAC, mean: 47.3 (11.7). Placebo: 46.3 (10.4)	97%	1 dose IM 1 g	8	Sleep quality VAS (1–100)	12	75/102 (73.5%)	High	Values of VAS not provided	No improvement in sleep quality with LAC	
Amitriptyline	Braz *et al.* 2013 [21]	Single site in Brazil	33	Median 43.2 (range 27–58)	100%	25 mg	12	Sleep quality VAS 0–10	12	26/33 (78.8%)	Some concerns	Sleep quality VAS (0–10): Amitriptyline (−3.6); Placebo (−4.5) [NS between groups]	No improvement in sleep quality with amitriptyline	
Amitriptyline	Carette *et al.* 1995 [53]	Single site in Canada	22	Mean 43.8 (8.0)	95.50%	25 mg	8	Sleep quality VAS (0–10); PSG	8	20/22 (90.9%)	High	Sleep quality VAS (0–10): Amitriptyline (−3.96); Placebo (−0.98) [*P*< 0.05]. PSG: TST, h: AMI (+0.42); Placebo (+0.14). Stage 1,%: AMI (+0.69); Placebo (−1.9). Stage 2,%: AMI (+5.16); Placebo (+0.94). Stage 3,%: AMI (−0.58); Placebo (+0.45). Stage 4,%: AMI (−2.6); Placebo (+0.09). Latency sleep, min: AMI (−2.76); Placebo (−10.04). Latency stage 3, min: AMI (−2.76); Placebo (−10.04). Latency stage 4, min: AMI (+1.41); Placebo (+0.2). Latency REM, min: AMI (+2.85); Placebo (−5.68). Stage 2 alpha, rating: AMI (+0.15); Placebo (−0.12). Stage 3 alpha, rating: AMI (−0.03); Placebo (+0.12). Stage 4 alpha, rating: AMI (+0.05); Placebo (+0.23). Stage 3 or 4 alpha, rating: AMI (+0.05); Placebo (+0.08)	No improvement in sleep quality with amitriptyline. On PSG, increase in stage 2 NREM sleep and decreased sleep latency with amitriptyline	Crossover trial
Amitriptyline	Ginsberg *et al.* 1996 [64]	Single site in France	51	Mean: 46 (12)	83%	25 mg	8	Sleep quality VAS (0–10)	8	46/51 (90.2%)	High	Sleep quality VAS (0–10): Amitriptyline (−2.6); Placebo (−0.3)	Improvement in sleep quality with amitriptyline	
Amitriptyline	Kempenaers *et al.* 1994 [54]	Single site in Belgium	24	Mean: 38 (7)	100%	50 mg	8	Sleep quality VAS (0–100). PSG	8	14/24 (58.3%)	High	Sleep quality VAS (0–100): AMI (+17); Placebo (+2). PSG: TST, min: AMI (−22); Placebo (+40) [NS]. Sleep onset latency, min: AMI (+6); Placebo (−3) [NS]. Sleep efficiency,%: AMI (−6.7); Placebo (+2) [NS]. Arousal index, n/h: AMI (+3); Placebo (+2) [*P* = 0.029]. Stage shifts index, n/h: AMI (+5); Placebo (−3) [NS]. Stage 1, min: AMI (+10); Placebo (+4) [NS]. Stage 2, min: AMI (−3); Placebo (+37) [NS]. Stage 3, min: AMI (−14); Placebo (+1) [NS]. Stage 4, min: AMI (−17); Placebo (−4) [NS]. REM sleep, min: AMI (+1); Placebo (−1) [NS]. REM latency, min: AMI (+9); Placebo (−3) [NS]	Improvement in sleep quality but not PSG parameters with amitriptyline	
Amitriptyline	Carette *et al.* 1994 [17]	11 sites in Canada	126	Amitriptyline mean 44.1; placebo 47.1	92.90%	10–50 mg	24	Sleep quality VAS 0–100	24	98/126 (77.7%)	Some concerns	Detailed results not given	No improvement in sleep quality with amitriptyline	
Amitriptyline	Goldenberg *et al.* 1986 [18]	Single site in USA	62	Median: 43.8 (21–69)	95.20%	25 mg	6	Sleep quality VAS 0–10	6	58/62 (93.5%)	Some concerns	Sleep quality VAS (0–10): Amitriptyline (−3.2); Amitriptyline + Naproxen (−2.0). Placebo values not given	Improvement in sleep quality with amitriptyline	
Amitriptyline	Goldenberg *et al.* 1996 [19]	Single site in USA	31	Mean: 43.2 (9.1)	90.30%	25 mg	6	Sleep quality VAS 0–100	6	19/31	Some concerns	Sleep quality VAS (0–100): Amitriptyline (−15.2); Amitriptyline + Fluoxetine (−34.7); Placebo (+6.6). Overall amitriptyline effect, *P* < 0.001	Improvement in sleep quality with amitriptyline. More marked with fluoxetine combination	Cross-over trial
Amitriptyline	Hannonen *et al.* 1998 [20]	Single site in Finland	97	Amitriptyline, mean: 49.7 (8.2); placebo: 48.9 (8.9)	100%	25–37.5 mg	12	Sleep quality VAS 0–10	12	62/97 (63.9%)	High	Sleep quality VAS (0–10): Amitriptyline −2.3; placebo −0.7	Improvement in sleep quality with amitriptyline	
Antidiencephalon Immunoglobulin (SER282)	Kempenaers *et al.* 1994 [54]	Single site in Belgium	24	Mean: 38 (7)	100%	20 mg/ml	8	Sleep quality VAS (0–100). PSG	8	17/24 (70.8%)	High	Sleep quality VAS (0–100): SER282 (−27); Placebo (+2). PSG: TST, min: SER282 (+6); Placebo (+40) [NS]. Sleep onset latency, min: SER282 (−3); Placebo (−3) [NS]. Sleep efficiency,%: SER282 (+5.2); Placebo (+2) [NS]. Arousal index, n/h: SER282 (+0); Placebo (+2) [*P* = 0.029]. Stage shifts index, n/h: SER282 (+2); Placebo (−3) [NS]. Stage 1, min: SER282 (+6); Placebo (+4) [NS]. Stage 2, min: SER282 (+2); Placebo (+37) [NS]. Stage 3, min: SER282 (−4); Placebo (+1) [NS]. Stage 4, min: SER282 (+13); Placebo (−4) [NS]. REM sleep, min: SER282 (−10); Placebo (−1) [NS]. REM latency, min: SER282 (−38); Placebo (−3) [NS]	No improvement in sleep quality or PSG parameters for SER282	
Carisoprodol/paracetamol/caffeine	Vaerøy *et al.* 1989 [55]	Single site in Norway	58	Mean: 47.7 (13.7)	100%	1200 mg/1920 mg/384 mg	8	Sleep quality VAS (0–10)	8	43/58 (74.1%)	High	Sleep quality VAS (0–10): treatment (+3.3); placebo (+2.7). [NS]	No improvement in sleep quality with treatment	
Citalopram	Nørregaard *et al.* 1995 [56]	Single site in Denmark	42	Citalopram, mean: 48 (9); placebo: 50 (9)	Not given	20–40 mg	8	Sleep quality NRS (0–10) on FIQ	8	33/42 (78.6%)	High	FIQ, sleep quality NRS (0–10): Citalopram (+1.0); Placebo (+0.1) [NS]	No improvement in sleep quality with citalopram	
Cyclobenzaprine	Carette *et al.* 1994 [17]	11 sites in Canada	124	CBP: mean 43.4; placebo 47.1	94.40%	10–30 mg	24	Sleep quality VAS 0–100	24	86/124 (69.4%)	Some concerns	Detailed results not given	No improvement in sleep quality with cyclobenzaprine	
Cyclobenzaprine	Moldofsky *et al.* 2011 [22]	2 sites in Canada	36	CBP, mean: 45.9 (11.4); placebo: 39.3 (9.3)	97%	Up to 4 mg	8	PSG	8	36/36 (100%)	Low	TST, h: CBP (+0.7); Placebo (−0.1) [NS]. Sleep efficiency,%: CBP (+11.5); Placebo (+3.0) [NS]. REM,%: CBP (−2.9); Placebo (+1.0) [*P* = 0.007]. Stage 1,%: CBP (+1.2); Placebo (−0.4) [NS]. Stage 2,%: CBP (+3.5); Placebo (−2.6) [*P* = 0.021]. Stage 3,%: CBP (+1.2); Placebo (−0.2) [NS]. Stage 4,%: CBP (−3.0); Placebo (+2.5) [*P* = 0.029]	Improvement in restorative sleep, increase in stage 2 and decrease in stage 4 and REM sleep with cyclobenzaprine	
Duloxetine	Gilron *et al.* 2016 [40]	Single centre in Canada	41	Median: 56 (20–71)	88%	Up to 120 mg	24	MOS-SS	24	33/41 (80.4%)	Low	MOS-SS SPI-1: Duloxetine (−1.0), Placebo (+1.8) MOS-SS SPI-2: Duloxetine (−1.0), Placebo (+0.5)	No improvement in MOS-SS sleep problem indices 1 and 2 with duloxetine	Cross-over trial. Four periods of 6 weeks on each drug
Esreboxetine	Arnold *et al.* 2010 [23]	56 centres in USA	268	Esreboxetine: median 49.2 (21–79); placebo 50.1 (20–84)	89.50%	2–8 mg	8	MOS-SS	8	213/268 (79.8%)	Some concerns	MOS-SS sleep problems index (0–100): Esreboxetine (−5.47); Placebo (−3.96) [NS]	No improvement in sleep quality on MOS-SS SPI with esreboxetine. Improvement in somnolence, quantity of sleep and snoring sub-scales	
Fluoxetine	Goldenberg *et al.* 1996 [19]	Single site in USA	31	Mean: 43.2 (9.1)	90.30%	20 mg	6	Sleep quality VAS 0–100	6	19/31	Some concerns	Sleep quality VAS (0–100): Fluoxetine (−8.6); Amitriptyline + Fluoxetine (−34.7). (Overall fluoxetine effect, *P* = 0.04)	Improvement in sleep quality with fluoxetine	Cross-over trial
Fluoxetine	Wolfe *et al.* 1994 [24]	Single site in USA	42	Fluoxetine, mean: 48 (10.1); placebo: 52.9 (11.3)	100%	20 mg	6	Sleep quality VAS (0–15)	6	24/42 (58.5%)	Some concerns	Sleep quality VAS (0–15): Fluoxetine (−2.0); Placebo (−2.0) [NS]	No improvement in sleep quality with fluoxetine	
Gabapentin	Arnold *et al.* 2007 [25]	3 sites in USA	150	Gabapentin: mean 49.2 (SD 10.6); placebo: 47.3 (11.8)	90%	Up to 1200 mg	12	MOS-SS	12	119/150 (79.3%)	Some concerns	MOS-SS sleep problems index (0–100): Gabapentin (−22.6); Placebo (−8.0) [*P*= 0.001]	Improvement in sleep quality with gabapentin	
Milnacipran	Ahmed *et al.* 2016 [26]	Single site in USA	19	Median 49.2 (range, 28–72)	89.50%	100 mg	12	MOS-SS sleep problem index 2; PSG; sleep quality NRS (0–10)	14	15/19 (78.9%)	Low	MOS-SS sleep problems index 2 (0–100); Milnacipran (−17.8); Placebo (−20.7) [NS]. Sleep quality NRS (0–10): Milnacipran (+1.1); Placebo (+0.8) [NS]. PSG: WASO, min: Milnacipran (−21); Placebo (−43.6) [NS]. TST, min: Milnacipran (+21); Placebo (+45.4) [NS]. Sleep efficiency,%: Milnacipran (+4.8); Placebo (+11) [NS]	No improvement in sleep quality with milnacipran	Cross-over trial, 6 weeks on each drug with 1 week washout
Milnacipran	Branco *et al.* 2010 [27]	89 sites in 13 European countries	884	Placebo: mean 49.2 (10.3), milnacipran: 48.3 (9.3)	Placebo 93.5%; milnacipran 95.1%	200 mg	17	MOSS-SS sleep problems index. Weekly sleep recall VAS (0–100)	16	678/884 (76.7%)	Some concerns	MOS-SS sleep problems index 1 (0–100): Milnacipran −6.28; Placebo −6.73; Milnacipran *vs* Placebo: 0.45 (NS). MOS-SS sleep problems index 2 (0–100): Milnacipran −6.93; Pregabalin −7.40; Milnacipran *vs* Placebo: 0.47 (NS). Weekly sleep recall VAS (0–100): Milnacipran −13.86; Placebo −9.59; Milnacipran *vs* Placebo −4.27 (*P* = 0.007)	Improvement in weekly sleep recall with milnacipran, but no improvement in MOS-SS	4 weeks dose escalation, 12 weeks stable dose, 9 weeks dose down-titration
Milnacipran	Clauw *et al.* 2008 [28]	86 centres in USA	1196	Placebo: mean 50.7 (10.4); 100 mg 49.5 (10.9); 200 mg 50.4 (10.6)	96.20%	100 mg, 200 mg	15	MOS-SS sleep problems index 2	15	811/1196 (67.8%)	Some concerns	MOS-SS sleep problems index 2 (0–100): Milnacipran 100 mg +1.7; Milnacipran 200 mg +2.3; Placebo +3.0 (all NS)	No improvement in sleep quality with milnacipran	6-month extension study
Milnacipran	Gendreau *et al.* 2005 [29]	14 sites in USA	125	Mean 47.0 (11.1)	98%	200 mg	13	JSS	12	90/125 (72%)	Some concerns	No significant change in JSS. Detailed results not reported	No improvement in sleep quality with milnacipran	
Milnacipran	Mease *et al.* 2009 [30]	59 sites in USA	888	Placebo, mean: 49.4 (10.1); 100 mg: 49.9 (10.6); 200 mg: 49.2 (11)	95.60%	100 mg, 200 mg	27	MOS-SS	27	512/888 (57.7%)	Some concerns	MOS-SS sleep problems index 1 (0–100): Milnacipran 100 mg +0.12; Milnacipran 200 mg −1.65; Placebo −0.06 (all NS). MOS-SS sleep problems index 2 (0–100): Milnacipran 100 mg −0.43; Milnacipran 200 mg −2.11; Placebo −0.96 (all NS)	No improvement in sleep quality with milnacipran	3 weeks dose escalation, 24 weeks stable dose
Milnacipran	Vitton *et al.* 2004 [57]	14 sites in USA	125	46.2–48.0 (group values not given)	96–98%	≤200 mg, 400 mg	12	JSS	12	90/125 (72%)	Some concerns	JSS (0–20): Milnacipran 200 mg (−1.3); 400 mg (−1.3); Placebo (−0.5). [NS]	No improvement in sleep quality with milnacipran	
Mirtazapine	Yeephu *et al.* 2013 [31]	Single site in Thailand	40	Mean: 44.7 (10.8)	100%	15 mg, 30 mg	13	JSS	13	32/40 (80%)	Some concerns	JSS (0–20): Mirtazapine 15 mg (−5.92); 30 mg (−6.54); Placebo (−2.8). [NS]	No improvement in sleep quality with mirtazapine	
Moclobemide	Hannonen *et al.* 1998 [20]	Single site in Finland	98	Moclobemide 47.6 (8.7); placebo 48.9 (8.9)	100%	450–600 mg	12	Sleep quality VAS 0–10	12	60/98 (61.2%)	High	Sleep quality VAS (0–10): Moclobemide −0.0; placebo −0.7	No improvement in sleep quality with moclobemide	
Naltrexone	Younger *et al.* 2013 [32]	USA	31	Mean: 42.7 (12.9)	100%	4.5 mg	12	Sleep quality VAS 0–100	12	28/31 (90.3%)	Some concerns	Sleep quality VAS (0–100), % improvement: Naltrexone (+10.4); Placebo (+9.2) [NS]	No improvement in sleep quality with naltrexone	Cross-over study
Panax Ginseng	Braz *et al.* 2013 [21]	Single site in Brazil	36	Median 43.2 (range 27–58)	100%	100 mg	12	Sleep quality VAS 0–10	12	25/36 (69.4%)	Some concerns	Sleep quality VAS (0–10): P Ginseng (−3.8); Placebo (−4.5) [NS between groups]	No improvement in sleep quality with panax ginseng	
Paroxetine	Giordano *et al.* 1999 [58]	Single site in Italy	40	Mean: 31 (7.2)	100%	20 mg	12	Sleep quality VAS 0–10	12	29/40 (72.5%)	High	Values of VAS not indicated	Improvement in sleep quality with paroxetine, but values not provided	
Pregabalin	Arnold *et al.* 2008 [33]	84 centres in USA	750	Mean 50.1 (11.4)	94.50%	300 mg, 450 mg, 600 mg	14	MOS-SS; sleep quality NRS (0–10)	14	600 mg 60.1%, 450 mg 66.8%, 300 mg 67.2%, placebo 67.9%	Low	MOS-SS SPI (0–100): 300 mg (−11.39), 450 mg (−12.85), 600 mg (−15.09) *vs* placebo (−6.65). Sleep quality NRS (0–10): 300 mg (−1.75); 450 mg (−2.03), 600 mg (−2.05), Placebo (−1.04)	Improvement in sleep quality with all pregabalin doses	
Pregabalin	Crofford *et al.* 2005 [34]	40 centres in USA	530	450 mg: mean 48.9 (11.3); 300 mg: 47.7 (10.1); 150 mg: (10.4); placebo: 49.7 (10.7)	91.30%	150 mg, 300 mg, 450 mg	8	MOS-SS; sleep quality NRS (0–10)	8	410/530 (77.3%); placebo 74%; 150 mg 78%; 300 mg 82.8%; 450 mg 75%	Some concerns	MOS-SS sleep problems index (0–100): 150 mg (−16.84); 300 mg (−17.24), 450 mg (−22.06), Placebo (−8.34). Sleep quality NRS (0–10): 150 mg (−1.69); 300 mg (−1.92), 450 mg (−2.61), Placebo (−1.3)	Improvement in sleep quality with all pregabalin doses	
Pregabalin	Crofford *et al.* 2008 [35]	95 sites in USA	566	Placebo: mean 49.6 (10.5); pregabalin: 48.8 (11.9)	90.10%	300 mg, 450 mg and 600 mg	26	MOS-SS sleep problems index	26	162/566 (28.6%)	Some concerns	MOS-SS sleep problems index (0–100): 300 mg (−40.2); 450 mg (−42.5), 600 mg (−39), Placebo (−40.4). Sleep quality NRS (0–10): 300 mg (−1.69); 450 mg (−1.92), 600 mg (−2.61), Placebo (−1.3)	No significant improvement in sleep quality with all pregabalin doses	
Pregabalin	Mease *et al.* 2008 [36]	79 sites in USA	751	600 mg, mean: 48.7 (11.2); 450 mg: 47.7 (10.8); 300 mg: 50.1 (10.4); placebo 48.6 (11.3)	94.40%	300 mg, 450 mg, 600 mg	13	MOS-SS; sleep quality NRS (0–10)	14	485/751 (64.8%)	Some concerns	MOS-SS sleep problems index (0–100): 300 mg (−19.1); 450 mg (−20.41); 600 mg (−19.49); Placebo (−14.29). Sleep quality NRS (0–10): 300 mg (−2.19); 450 mg (−2.29); 600 mg (−2.53); placebo (−1.32)	Improvement in sleep quality with pregabalin	
Pregabalin	Ohta *et al.* 2012 [37]	44 sites in Japan	501	Pregabalin, mean: 47.9 (12); placebo: 46.7 (12.6)	88.90%	150–450 mg	15	MOS-SS; NRS 0–10 (secondary outcomes)	15	415/501 (82.8%)	Some concerns	MOS-SS sleep problems index (0–100): Pregabalin (−10.3); Placebo (−6.14). Sleep quality NRS (0–10): Pregabalin (−1.52); Placebo (−0.79)	Improvement in sleep quality with pregabalin	
Pregabalin	Pauer *et al.* 2011 [38]	73 sites internationally	736	Mean: 48.5 (11.2)	91.00%	300 mg, 450 mg, 600 mg	14	MOS-SS; NRS 0–10 (secondary outcomes)	14	518/738 (70.2%)	Some concerns	MOS-SS sleep problems index (0–100): 300 mg (−13.18); 450 mg (−19.26); 600 mg (−18.7; placebo (−5.99). Sleep quality NRS (0–10): 300 mg (−1.45); 450 mg (−1.72); 600 mg (−1.95); placebo (−0.94)	Improvement in sleep quality with pregabalin	*Denmark, 2 centres, France 5, Germany 5, Italy 6, Portugal 4, Spain 4, Sweden 4, Switzerland 3, The Netherlands 5, UK 5, Australia 4, Canada 12, India 4, Korea 3, Mexico 4, Venezuela 3*
Pregabalin	Roth *et al.* 2012 [39]	19 sites in USA, Germany and Canada	119	Median 48.4 (range 27–77)	86.60%	300–450 mg	4	Sleep quality NRS (0–10); sleep diary; PSG	4	103/119 (85.7%)	Some concerns	Sleep quality NRS (0–10): Pregabalin *vs* Placebo: +0.89. PSG: WASO, min: Pregabalin (−56.8); placebo (−37.6). TST, min: Pregabalin (+72); Placebo (+50). Sleep efficiency,%: Pregabalin (+15.8); Placebo (+10.4). Latency to persistent sleep: Pregabalin (−24.6); Placebo (−17.5). Slow wave sleep, %: Pregabalin (+1.9); Placebo (−0.3)	Improvement in sleep quality with pregabalin	Cross-over trial; 2 weeks on each drug
Pregabalin	Gilron *et al.* 2016 [40]	Single centre in Canada	41	Median: 56 (20–71)	88%	Up to 450 mg	24	MOS-SS	24	33/41 (80.4%)	Low	MOS-SS SPI-1: Pregabalin (−11.9), Placebo (+1.8) MOS-SS SPI-2: Pregabalin (−13.1), Placebo (+0.5)	Improvement in MOS-SS sleep problem indices 1 and 2 with pregabalin and pregabalin/duloxetine combination	Cross-over trial. Four periods of 6 weeks on each drug
Pregabalin/Duloxetine	Gilron *et al.* 2016 [40]	Single centre in Canada	41	Median: 56 (20–71)	88%	450 mg/120 mg	24	MOS-SS	24	33/41 (80.4%)	Low	MOS-SS SPI-1: Pregabalin–Duloxetine (−15.0), Placebo (+1.8) MOS-SS SPI-2: Pregabalin–Duloxetine (−15.8), placebo (+0.5)	Improvement in MOS-SS sleep problem indices 1 and 2 with pregabalin/duloxetine combination	Cross-over trial. Four periods of 6 weeks on each drug
Quetiapine	Potvin *et al.* 2012 [41]	Single site in Canada	51	Quetiapine, mean: 50.0 (11.7); placebo: 49.1 (8.7)	100%	50–300 mg	12	PSQI	12	43/51 (84.3%)	Some concerns	PSQI (0–21): Quetiapine (−3.5); Placebo 0 [*P* = 0.009]	Improvement in sleep quality with quetiapine XR	
Ritanserin	Olin *et al.* 1998 [59]	Single site in Sweden	54	Median: 44 (24–59)	100%	10 mg	16	Sleep quality VAS (0–10)	16	51/54 (94.4%)	Some concerns	No detailed results given	No improvement in sleep quality with ritanserin	
Sodium Oxybate	Moldofsky *et al.* 2010 [42]	21 sites in USA	195	Mean: 46.5 (11.3)	93.80%	4.5 g, 6 g	8	JSS; PSG	8	151/195 (77.4%)	Some concerns	JSS (0–20): SXB 4.5.g (−7.1), 6 g (−8.4); placebo (−3.8). PSG WASO (min): 4.5 g (4.2); 6 g (−39.7); placebo (16.2). PSG TST (min): 4.5 g (−11.4); 6 g (39.4); Placebo (10.1). PSG sleep onset latency (min): 4.5 g (3.2); 6 g (−5.9); placebo (0.9). PSG sleep efficiency (%): 4.5 g (−2.4); 6 g (8.9); placebo (3.0). PSG REM sleep (min): 4.5 g (−23.8); 6 g (−18.6). PSG NREM sleep (min): 4.5 g (12.4); 6 g (57.9); placebo (12.4)	Improvement on subjective sleep quality with both doses of sodium oxybate. Increased NREM and decreased REM sleep with sodium oxybate	
Sodium Oxybate	Russell *et al.* 2009 [43]	21 sites in USA	195	Placebo, mean: 47.3 (10.6); 4.5 g: 47.4 (12.1); 6 g: 45.5 (11.6)	94.60%	4.5 g, 6 g	8	JSS	8	147/195	Some concerns	JSS (0–20): SXB 4.5.g (−5.5), 6 g (−6.0); placebo (−1.0)	Significant improvement in sleep quality with both doses of sodium oxybate	
Sodium Oxybate	Russell *et al.* 2011 [44]	74 clinical sites in USA	548	Mean: 47.0 (11.3)	91.20%	4.5 g, 6 g	14	JSS	16	334/548 (60.9%)	Some concerns	JSS (0–20): SXB 4.5.g (−6.1), 6 g (−6.2); placebo (−2.9)	Improvement in sleep quality with both doses of sodium oxybate	
Sodium Oxybate	Spaeth *et al.* 2012 [45]	108 centres internationally	578	Mean: 46.6 (10.7)	89.50%	4.5 g, 6 g	14	JSS	14	376/573 (65.6%)	Some concerns	JSS (0–20): Sodium Oxybate 4.5 g (−4.0); 6 g (−5.0); Placebo (−1.0). (all significant *vs* placebo)	Improvement in sleep quality with both doses of sodium oxybate	France, Germany, Italy, Netherlands, Spain, UK, USA
Tramadol/Acetaminophen	Bennett *et al.* 2003 [60]	Multicentre in USA	315	Mean 50 (10)	94%	37.5 mg/325 mg	13	Sleep index 6; sleep index 9	13	313/315 (99.4%)	High	Sleep index 6 (0–100): Tramadol (−8); Placebo (−8). Sleep index 9 (0–100): Tramadol (−7); Placebo (−7). [NS]	No improvement in sleep quality with tramadol/acetaminophen combination	
Zopiclone	Drewes *et al.* 1991 [46]	Single site in Denmark	45	Mean 50	100%	7.5 mg	12	PSG; Leeds Sleep Evaluation Questionnaire (VAS); Spiegel sleep questionnaire	12	41/45 (91.1%)	Some concerns	LSEQ: sleep onset latency (0–100): Zopiclone (+15.3); placebo (+3.8) [*P* < 0.05]. Quality of sleep (0–100): Zopiclone (+14.5); Placebo (+1.7) [*P* < 0.05]. Pattern of awakening: Zopiclone (+2.9); placebo (+0.2) [NS]. Feeling on waking: Zopiclone (−3.2); placebo (−6.3) [NS]. Feeling now: Zopiclone (−4.5); Placebo (−6.0) [NS]. Balance and coordination: Zopiclone (+1.9); placebo (−0.4) [NS]. Spiegel sleep questionnaire (1–5): sleep onset latency: Zopiclone (−0.9); placebo (−0.2) [*P* < 0.05]. Quality of sleep: Zopiclone (−1.0); placebo (−0.6) [*P* < 0.05]. Duration of sleep: Zopiclone (−0.9); placebo (+0.2) [*P* < 0.05]. Awakenings at night: Zopiclone (−0.8); placebo (−0.2) [*P* < 0.05]. Dreams: Zopiclone (0.5); placebo (−0.1) [NS]. Condition in the morning: Zopiclone (−0.7); Placebo (−0.4) [*P* < 0.05]. PSG: NREM stage 1,%: Zopiclone (−2.1); Placebo (−1.5); NREM stage 2,%: Zopiclone (+11.9); Placebo (+9.0); NREM stages 3 and 4,%: Zopiclone (−5.3); Placebo (−5.7). REM, %: Zopiclone (−2.9); Placebo (+5.1). Number of awakenings: Zopiclone (−24); Placebo (−4). Number of stage shifts: Zopiclone (−12); Placebo (−7). [all PSG NS]	Improvement in self-reported sleep quality, but no differences in PSG findings, in zopiclone	

This table summarizes the characteristics, interventions, sleep outcome measures and key findings of pharmacological studies investigating sleep quality in fibromyalgia. Information includes study settings, sample sizes, participant demographics, treatment dosages and durations, sleep measurement tools, follow-up periods, completion rates, risk of bias and reported changes in sleep outcomes. Results are presented with statistical significance where available, highlighting both subjective (e.g. sleep scales) and objective (e.g. polysomnography) measures of sleep quality. The findings highlight variable effects of pharmacological interventions on sleep quality, with some studies showing significant improvements and others reporting no notable differences. Methodological details, such as crossover designs and specific biases, are noted where relevant. LAC: acetyl L-carnitine; MOS-SS: Medical Outcomes Study Sleep Scale; NRS: numerical rating scale; PSG: polysomnography; JSS: Jenkins Sleep Scale; PSQI: Pittsburgh sleep quality index; VAS: visual analogue scale; FIQ: fibromyalgia impact questionnaire; WASO: wake after sleep onset; TST: total sleep time; NS: not significant; mg: milligrams.

**Table 2.** Characteristics and outcomes of cognitive behavioural therapy interventions for sleep quality in fibromyalgia

Intervention	Study	Setting	Randomized, N	Age, years	% Female	Control	Treatment duration	Sleep measure(s)	Follow-up, weeks	Completed study, %	Risk of bias	Change from baseline	Overall result
CBT-I	Edinger *et al.* 2005 [48]	Single site in USA	29	48.6 (8.2)	96.60%	TAU	1 session/week for 6 weeks. First session 45–60 min, subsequent session 15–30 min	Insomnia symptom questionnaire; sleep diary; actigraphy; PSG	8	82.80%	High	**ISQ (0**–**100): CBT-I (**−**13.0); TAU (**−**0.4) [*P* < 0.05].**Sleep diary: **sleep efficiency,%: CBT-I (+7.4); TAU (+2.1). TWT, min: CBT-I (**−**43.3); TAU (**−**9.5).** TST, min: CBT-I (+11.4); TAU (+6.5). **SOL, min: CBT-I (**−**16.0); TAU (**−**2.7).** WASO, min: CBT-I (−32.8); TAU (−13.8). Actigraphy: sleep efficiency,%: CBT-I (+2.3); TAU (−0.2). TWT, min: CBT-I (−16.2); TAU (−2.3). TST, min: CBT-I (−10.8); TAU (−2.7). **SOL, min: CBT-I (**−**5.5); TAU (**−**2.7).** WASO, min: CBT-I (−9.7); TAU (−6.9)	Improvement in sleep quality with CBT-I. Improvement in self-reported sleep efficiency, TWT, SOL. Improvement in SOL on actigraphy
CBT-I	Martínez *et al.* 2014 [49]	Single site in Spain	64	47.6 (6.8)	100%	SH	90 min/week for 6 weeks	PSQI	6	89%	Some concerns	**PSQI (0–21): CBT-I (**−**3.97); SH (**−**1.45) [*P* < 0.05]**	Improvement in sleep quality with CBT-I. Not maintained after 6 months
CBT-I	McCrae *et al.* 2019 [50]	Single site in USA	76	CBT-I: 54.13 (11.03); WLC: 52.27 (11.19)	100%	WLC	50 min/week for 8 weeks	Sleep quality Likert scale (1–5); sleep diary; actigraphy; PSG	8	72.40%	Some concerns	**Sleep quality (**1–5**): CBT-I (+0.7); WLC (+0.19) [*P* < 0.05].** Sleep diary: SOL, min: CBT-I (−36.05); WLC (−20.16) [NS]. **WASO, min: CBT-I (**−**29.94); WLC (**−**10.45) [*P* < 0.05].** TST, min: CBT-I (+29.83); WLC (+32.52) [NS]. **Sleep efficiency,%: CBT-I (+15.12); WLC (+6.49) [*P* < 0.05].** PSG: SOL, min: CBT-I (−10.3); WLC (+1.38). **WASO, min: CBT-I (**−**19.35); WLC (+1.38).** TST, min: CBT-I (−10.98); WLC (−16.54). Sleep efficiency,%: CBT-I (+5.25); WLC (+1.25). Actigraphy: SOL, min: CBT-I (−13.98); WLC (−11.86). WASO, min: CBT-I (−6.76); WLC (−0.99). TST, min: CBT-I (−16.38); WLC (−8.43). Sleep efficiency,%: CBT-I (+2.4); WLC (+1.21)	Improvement in sleep quality with CBT-I. Improvement maintained after 6 months. Less WASO on PSG in CBT-I *vs* control. No other significant differences in actigraphy or PSG measures
CBT-I	Miró *et al.* 2011 [47]	Single site in Spain	44	46.5 (7.03)	100%	SH	90 min/week for 6 weeks	PSQI	7	90%	High	**PSQI (0**–**21): CBT-I (**−**3.5); SH (**−**0.95) [*P* < 0.05]**	Improvement in sleep quality with CBT-I
CBT-I	Sánchez *et al.* 2012 [61]	Single site in Spain	26	46.79 (5.15)	100%	SH	90 min/week for 6 weeks	PSG	6	NI	High	PSG: TST, min: CBT-I (−9); SH (−34). WASO, min: CBT-I (−4); SH 0. Sleep efficiency,%: CBT-I (+3); SH (0.83). REM,%: CBT-I (−0.05); SH (+3.41). Stage 1,%: CBT-I (−2.34); SH (+3.41). Stage 2,%: CBT-I (−3.31); SH (−0.2). Stage 3,%: CBT-I (+2.97); SH (+0.12). Stage 4,%: CBT-I (+2.61); SH (−0.89). Arousals, N: CBT-I (+2.63); SH (−0.3). **Deep sleep: CBT-I (+5.55); SH (**−**0.79).** Light sleep (−5.69); SH (−2.67)	No increase in sleep time, but increase in time in deep sleep on PSG in CBT-I
CBT-IP	Castel *et al.* 2012 [62]	Single site in Spain	64	49.6 (6.8)	96.90%	TAU	2 h/week for 14 weeks (3 sessions of CBT-I)	MOS-SS	14	85.90%	High	**MOS-SS sleep problems index (0–100): CBT-IP (+9.4); Control (**−**0.1) [*P* < 0.001]**	Improvement in sleep quality with CBT-IP. Improvement maintained at 3- and 6-month follow-up
CBT-IP	Lami *et al.* 2018 [51]	Single site in Spain	84	50.2 (8.2)	100%	TAU	90 min/week for 9 weeks in groups of 5–7 people	PSQI	9	75%	High	PSQI (0–21): CBT-IP (−1.49); TAU (+0.2) [NS]	No improvement in sleep quality with CBT-IP
CBT-P	Lami *et al.* 2018 [51]	Single site in Spain	84	50.2 (8.2)	100%	TAU	90 min/week for 9 weeks in groups of 5–7 people	PSQI	9	76.20%	High	PSQI (0–21): CBT-P (+0.21); TAU (+0.2) [NS]	No improvement in sleep quality with CBT-P
CBT-P	McCrae *et al.* 2019 [50]	Single site in USA	74	CBT-P: 51.54 (10.62); WLC: 52.27 (11.19)	93.40%	WLC	50 min/week for 8 weeks	Sleep quality Likert scale (1–5); sleep diary; actigraphy; PSG	8	78.40%	Some concerns	**Sleep quality** (1–5)**: CBT-P (+0.49); WLC (+0.19) [*P* < 0.05].** Sleep diary: SOL, min: CBT-P (−25.0); WLC (−20.16) [NS]. WASO, min: CBT-P (−12.49); WLC (−10.45) [NS]. TST, min: CBT-P (+28.96); WLC (+32.52) [NS]. Sleep efficiency,%: CBT-P (+6.93); WLC (+6.49) [NS]. PSG: SOL, min: CBT-P (+0.69); WLC (+1.38). WASO, min: CBT-P (−24.76); WLC (+1.38). TST, min: CBT-P (+25.85); WLC (−16.54). Sleep efficiency,%: CBT-P (+5.91); WLC (+1.25). Actigraphy: SOL, min: CBT-P (−8.98); WLC (−11.86). WASO, min: CBT-P (−1.29); WLC (−0.99). TST, min: CBT-P (+2.36); WLC (−8.43). Sleep efficiency,%: CBT-P (+0.49); WLC (+1.21)	Improvement in sleep quality with CBT-P. Improvement maintained after 6 months. No significant differences in actigraphy or PSG measures
CBT-P	Saral *et al.* 2016 [63]	Single site in Turkey	66	Long term: 38.3 (9.8). Short term: 43.2 (9.2). Control: 43.7 (1.1)	100%	TAU	Long term: 180 min/week for 10 weeks. Short term: 2 190 min sessions	Sleep quality VAS (0–10)	26	89.40%	High	Sleep quality VAS (0–10): CBT-P LT (−4.2); CBT-P ST (−2.1); Control (−0.9)	No improvement in sleep quality with CBT-P

This table summarizes the study characteristics, treatment protocols, control conditions, sleep outcome measures and results of cognitive behavioural therapy interventions targeting sleep quality in fibromyalgia patients. Interventions include cognitive behavioural therapy for insomnia (CBT-I), pain (CBT-P) and combined CBT-I/CBT-P (CBT-IP), with comparisons against controls such as sleep hygiene (SH), treatment-as-usual (TAU) and waitlist controls (WLC). The primary sleep outcomes include the Pittsburgh sleep quality index (PSQI), visual analogue scale (VAS) for sleep quality, and polysomnography (PSG) measures such as total sleep time (TST), wake after sleep onset (WASO), and sleep onset latency (SOL). Statistically significant improvements are noted in **bold**. NI: not indicated; MOS-SS: Medical Outcomes Study Sleep Scale; TWT; Total Wake Time; ST: Short Term; LT: Long Term.

### Risk of bias

**Figure 2.**
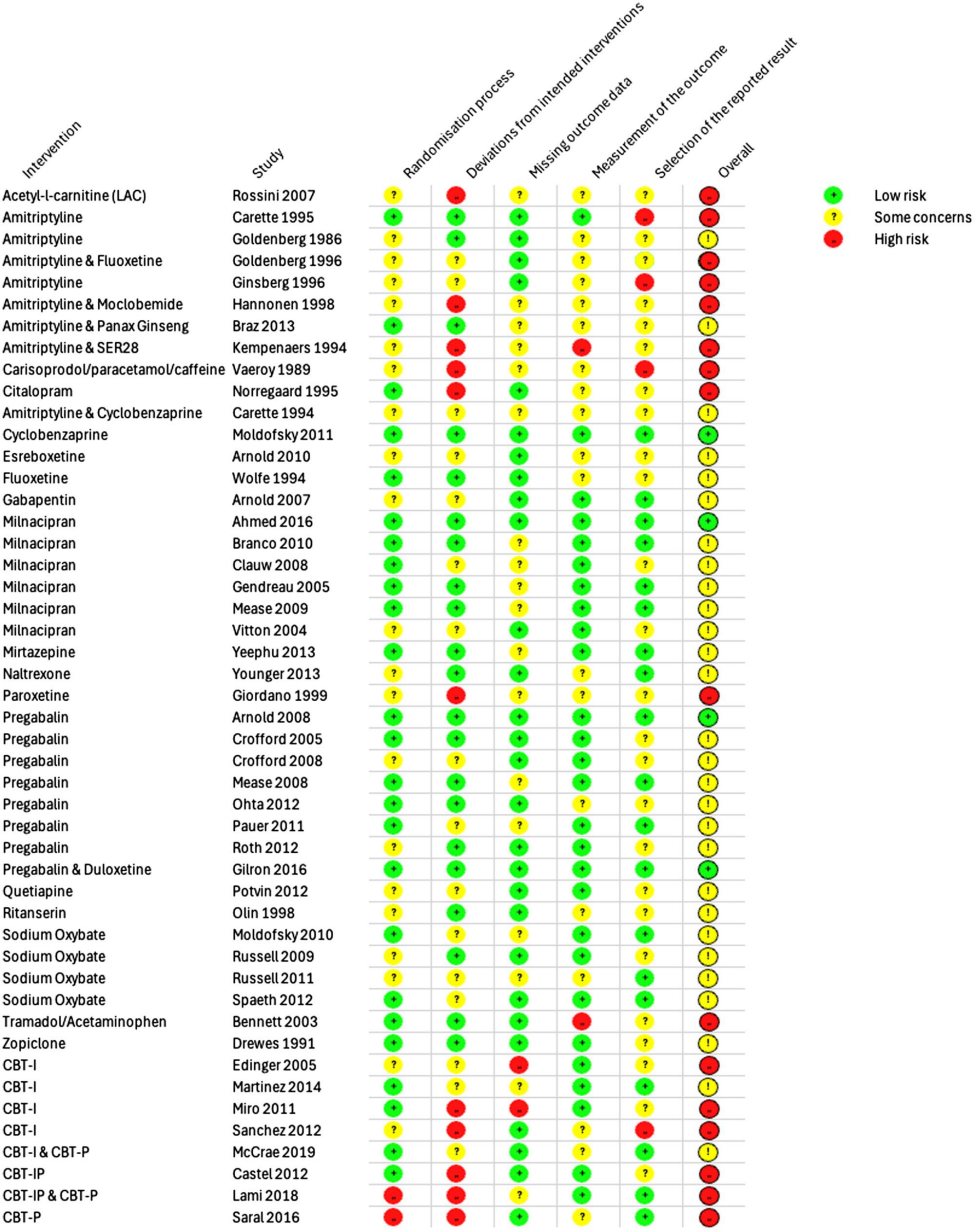
Risk of bias assessment for included studies. The figure summarizes the risk of bias across studies included in this systematic review, grouped by intervention type. Domains assessed include randomization process, deviations from intended interventions, missing outcome data, measurement of the outcome, selection of the reported result and overall risk of bias. Green circles represent ‘low risk’, yellow circles indicate ‘some concerns’, and red circles denote ‘high risk’ for each domain. The overall risk of bias for each study was determined by combining the judgements across all domains using the Cochrane Risk of Bias tool. Note, some studies evaluated multiple interventions. CBT-I: cognitive behavioural therapy for insomnia; CBT-P: cognitive behavioural therapy for pain; CBT-IP: combined CBT-I and CBT-P

Among 40 pharmacological studies, 10 (25%) were rated high risk [19, 20, 52–56, 58, 60, 64], 26 (66%) had some concerns [17–19, 21, 23–25, 27–32, 34–46, 57, 59], and 4 (10%) were low risk [22, 26, 33, 40] (Fig. 2). Pharmacological studies generally performed well in terms of randomization and adherence to treatment protocols, with over half rated low risk in these areas. Missing data and outcome measurement also showed reasonable levels of bias, with 24 (60%) studies low risk [18, 19, 22–26, 32–35, 37, 39–41, 43, 45, 46, 57, 59, 64], though selective reporting remained a concern, with only 15 (37.5%) studies rated low risk in this domain [22, 25–27, 29–33, 36, 38, 40, 42, 44, 45].

In contrast, CBT studies showed a higher overall risk of bias, with six (75%) rated high risk [47, 48, 51, 61–63] and none rated low risk (Fig. 2). Adherence to treatment protocols was poor, with five (62.5%) studies rated high risk for deviations from intended interventions [47, 51, 61–63]. Missing data and selective reporting were also problematic in CBT studies, possibly due to difficulties in maintaining participant engagement. Although randomization was reasonably strong, with four (50%) CBT studies rated low risk [47, 49, 50, 62], issues in treatment adherence, missing data and outcome measurement made achieving a low overall bias difficult. A detailed breakdown is given in Supplementary Fig. S1, available at *Rheumatology* online.

### Pharmacological interventions

#### Gabapentinoids

##### Pregabalin

**Figure 3.**
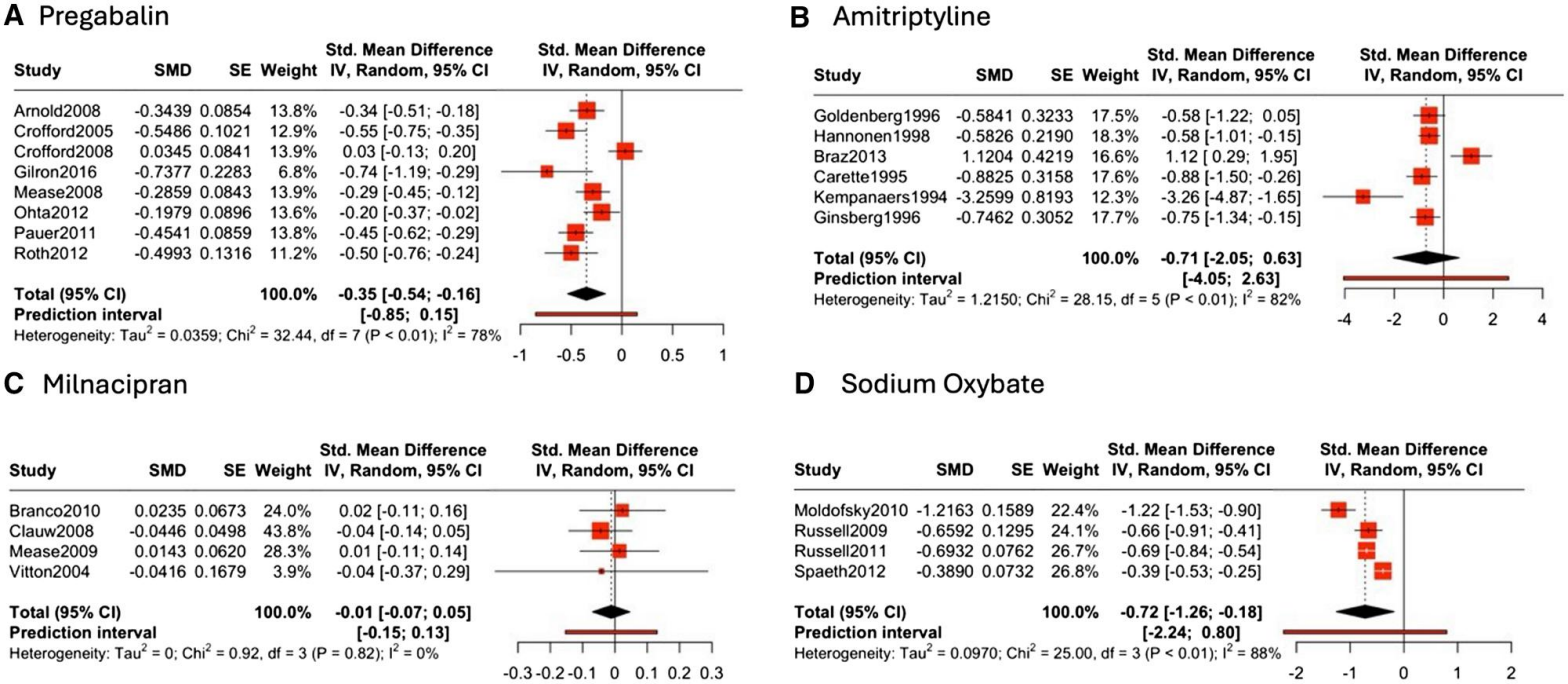
Forest plot of the effect of pharmacological interventions on sleep quality in fibromyalgia. The diamond at the bottom depicts the overall pooled effect, with its width representing the 95% CI. The size of each square corresponds to the weight of the study in the meta-analysis. For pregabalin (**A**), the pooled standardized mean differences (SMDs) and variances were calculated across all doses evaluated in each study, with crossover studies (e.g. Gilron *et al*. [40], Roth *et al*. [39]) appropriately handled using variance reduction techniques. Amitriptyline (**B**) utilized baseline-adjusted standardized mean difference (SMD) to account for pre-treatment differences between intervention and control groups. For Braz *et al*. [21], baseline means and SDs were estimated from medians and 95% CIs using the methods of Luo *et al*. [65] and Wan *et al*. [66]. Follow-up means were derived from reported percentage improvements, while follow-up SDs were imputed assuming a correlation of 0.7 between baseline and follow-up values. For sodium oxybate (**C**), SMDs for 4.5 and 6 g doses were combined using inverse variance weighting to produce a pooled effect. Milnacipran (**D**) SMDs were calculated based on the change scores from baseline to follow-up and pooled for studies with multiple doses. Hartung–Knapp (HK) adjustment was applied in all random-effects models to provide more accurate estimation of uncertainty

Eight studies examined pregabalin in 3834 patients using doses of 300, 450 and 600 mg over 4–26 weeks [33–40]. Gilron *et al.* [40] also evaluated pregabalin–duloxetine combination. A meta-analysis of pooled pregabalin doses showed a statistically significant moderate improvement on sleep quality (*n* = 8, SMD −0.35, 95% CI −0.54 to −0.16) (Fig. 3A). However, there was significant heterogeneity (t^2^ = 0.04, *P* < 0.01, I^2^ = 78%), and the prediction interval suggests some uncertainty about future studies, although a beneficial effect is likely. Crofford *et al.* [35] exerted the largest influence on the meta-analysis, but sensitivity analyses omitting this study did not meaningfully change the overall effect size. Sensitivity analyses showed a trend towards a dose–response relationship, with better results at 450 mg (SMD −0.43, 95% CI −0.71 to −0.16) than 300 mg (SMD −0.26, 95% CI −0.46 to −0.07), but no improvement in efficacy at 600 mg (SMD −0.29, 95% CI −0.72 to 0.14) (Supplementary Fig. S2, available at *Rheumatology* online). There was no evidence of publication bias detected based on Egger’s test (*P* = 0.18) (Supplementary Fig. S3, available at *Rheumatology* online).

##### Gabapentin

One study [25] investigated gabapentin doses of up to 1200 mg daily over 12 weeks in 150 patients and found a statistically significant improvement in sleep quality on the MOS-SS.

#### Antidepressants

##### Tricyclic antidepressants

Eight studies (*n* = 741) assessed amitriptyline (25–50 mg daily, 6–24 weeks) [17–21, 53, 54, 64], all using visual analogue scale (VAS) for sleep quality, with six included in the meta-analysis.

There was no statistically significant effect on sleep quality (Fig. 3B, n = 6, SMD −0.71, 95% CI −2.05 to 0.63), with significant heterogeneity observed (t^2^ = 1.22, *P* < 0.01, I^2^ = 82%). The wide prediction interval (−4.05 to 2.63) indicates uncertainty, which may reflect the small sample sizes and variable treatment durations.

Influence analysis identified Braz *et al.* [21] as contributing disproportionately to heterogeneity. Sensitivity analysis excluding this study strengthened the negative effect estimate (Supplementary Fig. S4, available at *Rheumatology* online, *n* = 5, SMD −0.75, 95% CI −1.37 to −0.13), reduced heterogeneity (I^2^ = 62%) and narrowed the prediction interval (−1.19 to −0.31), indicating greater consistency across studies after removing this outlier. Of two studies evaluating polysomnography (PSG) measures, one observed an improvement in sleep measures [53].

##### Selective serotonin reuptake inhibitors

Two studies investigated fluoxetine, of which one demonstrated improvement in sleep [19], and one did not [24]. A single study [56] investigated citalopram and did not find any improvement in sleep quality. Another study found a statistically significant improvement in sleep with paroxetine [58] although detailed results were not presented.

##### Serotonin-noradrenaline reuptake inhibitors

Six studies investigated milnacipran in 3237 patients [26–30, 57]. None found statistically significant improvement in sleep except Vitton *et al.* [57] which reported improved patient’s ability to stay asleep but did not report detailed outcome data. A meta-analysis of four studies showed no improvement in sleep quality (Fig. 3C, n = 4, SMD −0.01, 95% CI −0.07 to 0.05). There was low heterogeneity observed (t^2^ = 0, *P* = 0.82, I^2^ = 0%), with a narrow prediction interval suggesting reasonable certainty about this finding. There was no difference no dose–response relationship (Supplementary Fig. S5, available at *Rheumatology* online).

A single study investigated esreboxetine in 268 patients [23]. Although there was no improvement on the MOS-SS overall sleep problems index, there was an improvement in somnolence, snoring and quality of life MOS sub-scales.

One study evaluated duloxetine in 41 patients over 24 weeks and found no improvement in sleep quality [40]. The same study found that pregabalin–duloxetine combination did improve sleep quality on the MOS-SS sleep problems index compared with placebo but not compared with pregabalin alone.

##### Monoamine oxidase inhibitors

A single study investigated moclobemide in 98 patients and found no improvement in sleep quality on a VAS [20].

##### Atypical antidepressants

A single study of 40 patients [31] found that mirtazapine had no significant impact on sleep quality on the JSS compared with placebo.

#### Other agents

##### Sodium oxybate

Four studies investigated 4.5 and 6 g sodium oxybate daily over 8–16 weeks in 1516 patients [42–45]. All measured sleep quality using the JSS. Meta-analysis of pooled doses demonstrated a significant benefit on sleep quality (Fig. 3D, n = 4, SMD −0.72, 95% CI −1.26 to −0.18). There was significant heterogeneity observed (t^2^ = 0.097, *P* < 0.01, I^2^ = 88%), with a wide prediction interval reflecting considerable uncertainty in the result. Sensitivity analyses suggest a trend towards a dose–response relationship, with a larger effect with 6 g (SMD −0.72, 95% CI −1.39 to −0.06) compared with 4.5 g (SMD −0.54, 95% CI −0.93 to −0.15) (Supplementary Fig. S6, available at *Rheumatology* online). One study evaluated PSG and demonstrated increase time in non-rapid eye movement sleep (NREM) and decreased time in rapid eye movement sleep (REM) sleep in the sodium oxybate group compared with placebo [42].

##### Other pharmacological agents

One study of cyclobenzaprine demonstrated no improvement in sleep quality [17]; however, a smaller study of the same agent observed an increase in restorative sleep on PSG [22]. One study of 51 patients [41] found that quetiapine improved sleep quality on the PSQI. A small study of 45 patients found zopiclone improved self-reported sleep measures, but there were no differences in PSG measures [46].

No improvement in sleep quality was observed with naproxen [18], tramadol [60], panax ginseng [21], ritanserin [59], naltrexone [32], acetyl l-carnitine [52], carisoprodol with paracetamol and caffeine [55], and antidiencephalon immune serum [54].

### Cognitive behavioural therapy

#### CBT-I

Seven studies investigated CBT-I in 387 patients; five evaluated CBT-I alone (*n* = 239) [47–50, 61], while two studied CBT-I as part of a combined CBT-I/CBT-P programme (*n* = 148) [51, 62]. All CBT programmes were delivered by a clinician and lasted 6–14 weeks. A range of outcome measures were evaluated in relation to sleep, including the PSQI (*n* = 3), MOS-SS (*n* = 1), insomnia symptom questionnaire (*n* = 1), sleep quality Likert scale (*n* = 1) and PSG (*n* = 3). In contrast to the pharmacological studies evaluated, the majority of CBT intervention studies considered sleep-related outcome measures as primary outcomes. Three studies employed sleep hygiene as a control, while four used treatment as usual.

**Figure 4.**
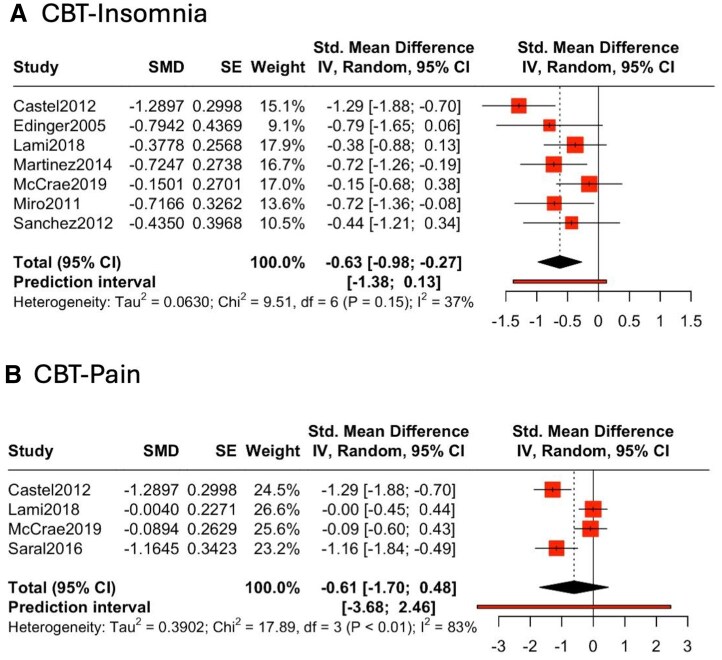
Forest plot of the effect of cognitive behavioural therapy for insomnia (CBT-I) on sleep quality in fibromyalgia patients. The SMD for each study is presented with corresponding standard errors (SEs) and 95% CIs. Effect sizes were adjusted to account for baseline differences in sleep quality measures between control and intervention groups. The diamond at the bottom of the plot represents the overall pooled effect, with its width corresponding to the 95% CI. The size of each square is proportional to the weight of the study in the random-effects model. The random-effects model uses the restricted maximum likelihood (REML) method to estimate between-study variance, with Hartung–Knapp (HK) adjustment applied to provide more robust estimates of uncertainty. SMD: standardized mean difference

A meta-analysis of CBT-I showed a statistically significant moderate improvement on sleep quality (*n* = 7, SMD −0.63, 95% CI −0.98 to −0.27) (Fig. 4A). There was moderate heterogeneity observed (t^2^ = 0.06, *P* = 0.15, I^2^ = 37%), with a relatively narrow prediction interval suggesting reasonable certainty regarding this finding. Sensitivity analyses show that Castel *et al.* [62] had the largest influence, but omitting this study did not affect the overall effect size. There was no evidence of publication bias on Egger’s test (*P* = 0.60) (Supplementary Fig. S7, available at *Rheumatology* online).

Of the studies that evaluated PSG, Edinger *et al.* [48] observed an improvement in sleep onset latency (SOL), while McCrae *et al.* [50] observed lower wake-after-sleep-onset (WASO), and Sánchez *et al.* [61] found an increase in time spent in deep sleep. Three studies continued follow-up beyond the treatment period, and two [50, 62] demonstrated that improvement in self-rated sleep quality was maintained after 6 months, while the other study [49] found that the effect was non-significant at this time.

#### CBT-P

Four studies evaluated CBT targeting pain in 288 patients [50, 51, 62, 63], with treatment duration ranging from 2 days to 26 weeks. Sleep outcome measures included PSQI (*n* = 1), MOS-SS (*n* = 1), sleep quality VAS or Likert scale (*n* = 2) and PSG (*n* = 1). All studies used treatment as usual or waiting list control. In contrast to CBT-I, meta-analysis showed no improvement in sleep with CBT-P (Fig. 4B, SMD −0.61, 95% CI −1.70 to 0.48). There was substantial heterogeneity observed (t^2^ = 0.39, *P* < 0.01, I^2^ = 83%), and a wide prediction interval suggesting considerable uncertainty.

## Discussion

### Summary

CBT-I is a promising intervention for improving sleep in fibromyalgia, while pregabalin and sodium oxybate may also be beneficial. In contrast, amitriptyline, milnacipran or CBT-P show no benefit. Other agents, such as gabapentin, quetiapine and zopiclone, demonstrate benefits in individual trials.

### Comparison with existing literature

#### Pharmacological agents

Although prior studies suggest amitriptyline improves sleep onset and continuity [67], we find limited evidence for improved sleep quality, aligning with findings in other pain conditions like diabetic neuropathy [68]. This contrasts with earlier reviews supporting amitriptyline’s sleep benefits [69]. High heterogeneity and a wide prediction interval highlight uncertainty. Sensitivity analysis revealed that the inclusion of Braz *et al.* [21] negated a significant pooled effect; its exclusion showed a statistically significant benefit, possibly explaining the overall lack of effect in our review.

Methodological limitations further complicate interpretation. Sleep assessments relied on VAS scales, which lack validation for chronic pain-related sleep quality, unlike MOS, JSS and PSQI [70]. In fibromyalgia, VAS scores correlate moderately with MOS sleep disturbance (r = 0.42–0.45) but more strongly with pain (r = 0.58–0.64), suggesting they may reflect pain levels rather than sleep quality [71–73]. Cognitive factors such as catastrophizing and functional interference [74] may further bias sleep ratings.

Pregabalin improves sleep across a range of clinical conditions [75] through direct effects rather than pain relief [76]. It enhances slow-wave sleep (SWS) which is particularly relevant to fibromyalgia, where SWS is reduced [77, 78]. However, concerns about adverse events and abuse, especially with opioids [79], have led NICE to withdraw its recommendation (NG193 [9]). While this review supports pregabalin’s sleep benefits, in line with previous reviews [80], these must be weighed against its misuse potential.

Sodium oxybate increases SWS and sleep continuity [81]. Although it may enhance sleep in fibromyalgia, the evidence is limited, and concerns over misuse limit its role.

Among other first-line treatments for fibromyalgia [82], duloxetine has the least evidence for improving sleep quality, in contrast to previous reviews [83]. Only one study met inclusion criteria, which found no significant benefit [40]. Studies using the Brief Pain Inventory (e.g. Arnold *et al.* [84, 85]) were excluded as they assess pain interference rather than sleep. The included study, Gilron *et al.* [40], found no benefit of duloxetine on sleep quality on the MOS-SS compared with placebo or pregabalin. Future research of duloxetine should use validated subjective and objective sleep measures.

#### CBT

This review finds a significant beneficial effect of CBT-I on sleep quality in fibromyalgia, updating prior findings [86], though limited by the high overall risk of bias. This aligns with evidence from other musculoskeletal pain disorders [87] and insomnia [88]. While CBT-I is a first-line treatment for insomnia, its impact on objective PSG sleep measures remains unclear [89], reinforcing the need for studies incorporating both subjective and objective assessments. Variability in CBT-I protocols, outcome measures and control arms complicates interpretation. Scaling CBT-I is challenging due to a shortage of trained clinicians, but digital CBT-I may offer a viable alternative with comparable effectiveness [90]. However, co-morbid symptoms like brain fog and fatigue may hinder engagement.

In contrast, CBT-P showed no significant effect on sleep. While it improves mood and pain coping in fibromyalgia [91, 92], its impact on sleep is minimal. This suggests that non-pharmacological sleep interventions should target sleep directly (i.e. CBT-I) rather than indirectly through mood or pain. Notably, combined CBT-I/CBT-P did not outperform CBT-I alone.

### Strengths and limitations

This review includes a comprehensive evaluation of both pharmacological and CBT interventions for sleep in fibromyalgia. It is one of few to evaluate the efficacy of both CBT-I and CBT-P, underscoring CBT-I’s specific benefits for sleep quality in fibromyalgia.

However, heterogeneity in inclusion criteria, fibromyalgia case definitions, intervention protocols and outcome measures limits comparability. Fibromyalgia diagnostic criteria evolved between 1990 and 2016, expanding beyond tender points to include fatigue, cognitive dysfunction and sleep disturbances, increasing population variability and potentially influencing baseline sleep quality and treatment responses.

Most pharmacological studies focused on pain rather than sleep, reducing specificity. CBT trials had heterogeneous controls and high bias risk, especially incomplete follow-up.

Sleep assessments varied, with subjective (e.g. MOS-SS, JSS, VAS/NRS) and objective (e.g. PSG, actigraphy) measures. Subjective measures may reflect perceived refreshment rather than true sleep quality, influenced by study focus, mood and cognitive biases [93]. Conversely, actigraphy and PSG provide objective sleep data, though self-reported measures remain clinically relevant, aligning with patient goals [94].

A median 12-week follow-up in pharmacological studies limits conclusions on long-term effects. However, three CBT-I trials followed participants for 6 months, with two demonstrating sustained benefits [49, 50, 62]. While this review focused on placebo-controlled trials for clearer intervention assessment, some studies compared treatments directly. For example, Gilron *et al.* [40] found a pregabalin–duloxetine combination superior to duloxetine alone but not pregabalin, while Lami *et al.* [51] reported greater sleep benefits with CBT-IP over CBT-P.

## Conclusion

This review reinforces CBT-I as a promising treatment for enhancing sleep quality in fibromyalgia, though further research is needed to identify the most effective modalities and doses. In contrast, CBT-P is unlikely to benefit sleep, though it may help with other symptoms.

Pharmacotherapy should not be a first-line approach to sleep disturbances in fibromyalgia. Amitriptyline and duloxetine lack consistent benefits, while pregabalin offers some efficacy but carries risks of misuse and should be used with caution. Future studies should assess combinations of pharmacotherapy and behavioural interventions, aiming to balance effectiveness with safety. A better understanding of fibromyalgia’s pathophysiology could guide drug development towards more targeted, low-risk treatments.

Stratifying patient populations by clinical characteristics could improve treatment outcomes. Future studies should use both validated self-reported sleep assessments alongside objective measures to better understand how sleep treatments work. Finally, research should go beyond pain and insomnia to assess broader impacts on quality of life [95].

## Supplementary material

Supplementary material is available at *Rheumatology* online.

## Supplementary Material

keaf147_Supplementary_Data

## Data Availability

The data underlying this article will be shared on reasonable request to the corresponding author.
