# Supplementary material for: Treatments for enhancing sleep quality in fibromyalgia: a systematic review and meta-analysis
Source: Rheumatology (Oxford). 2025 Mar 14;64(8):4495–516. doi: 10.1093/rheumatology/keaf147 (PMC12316380; doi:10.1093/rheumatology/keaf147)
Supplement: keaf147_Supplementary_Data [file keaf147_supplementary_data.docx]

# Supplementary Materials

## Supplementary Data S1. Search Strategy

**PubMed, MEDLINE, Embase,** **CINAHL, Cochrane CENTRAL and the International Trial Registries** (The World Health Organisations trials portal (ICTRP)) will be searched using the following search string for randomised controlled trials:

**PubMed, MEDLINE**

fibromyalgia[Title]) AND (sleep) AND (CBT OR cognitive behavioural therapy OR pharmacological OR drugs OR benzodiazepine OR sedating antidepressants OR sedating antihistamine or amphetamines OR SSRI OR SNRI)

**Embase**

1. Fibromyalgia*  :ab,ti,kw
2. Sleep* :ab, ti, kw
3. (CBT* OR cognitive behavioural therapy)
4. (pharmacological* OR drugs* OR benzodiazepine* OR sedating antidepressants* OR sedating antihistamine* OR amphetamines* OR SSRI* OR SNRI*) :ab,ti,kw
5. Randomized controlled trial* :pt
6. #5 or #6
7. #3 and #4 and #8 and #9

**CINAHL**

fibromyalgia[Title])

AND (sleep) [Abstract]

AND (CBT OR cognitive behavioural therapy OR pharmacological OR drugs OR benzodiazepine OR sedating antidepressants OR sedating antihistamine or amphetamines OR SSRI OR SNRI) [Title]

*Filters: articles*

**Cochrane CENTRAL**

Fibromyalgia [Title])

AND (sleep) [Title Abstract Keyword]

AND (CBT OR cognitive behavioural therapy OR pharmacological OR drugs OR benzodiazepine OR sedating antidepressants OR sedating antihistamine or amphetamines OR SSRI OR SNRI) [Title]

*Filters: trials*

**International trials registers**

(sleep) AND (CBT OR cognitive behavioural therapy OR pharmacological OR drugs OR benzodiazepine OR sedating antidepressants OR sedating antihistamine or amphetamines OR SSRI OR SNRI) | Completed Studies | Studies With Results | Interventional Studies | Fibromyalgia | Adult, Older Adult | Last update posted from 01/01/1980 to 01/02/2025

| **Study** | **PMID or DOI** | **NCT** |
| --- | --- | --- |
| Ahmed 2016 (Ahmed, Aamir et al. 2016) | PMID: 26414990 | NCT01234675 |
| Arnold 2007 (Arnold, Goldenberg et al. 2007) | DOI: 10.1002/art.22457 | Not available |
| Arnold 2008 (Arnold, Russell et al. 2008) | DOI: 10.1016/j.jpain.2008.03.013 | Not available |
| Arnold 2010 (Arnold, Chatamra et al. 2010) | PMID: 20974319 | NCT00357825 |
| Bennett 2003 (Bennett, Kamin et al. 2003) | DOI: 10.1016/s0002-9343(03)00116-5 | Not available |
| Branco 2010 (Branco, Zachrisson et al. 2010) | PMID: 20156949 | NCT00436033 |
| Braz 2013 (Braz, Morais et al. 2013) | DOI: 10.1016/j.rbp.2013.01.004 | Not available |
| Carette 1994 (Carette, Bell et al. 1994) | DOI: 10.1002/art.1780370106 | Not available |
| Carette 1995 (Carette, Oakson et al. 1995) | DOI: 10.1002/art.1780380906 | Not available |
| Clauw 2008 (Clauw, Mease et al. 2008) | PMID: 19108787 | NCT00098124 |
| Crofford 2005 (Crofford, Rowbotham et al. 2005) | DOI: 10.1002/art.20983 | Not available |
| Crofford 2008 (Crofford, Mease et al. 2008) | DOI: 10.1016/j.pain.2008.02.027 | Not available |
| Drewes 1991 (Drewes, Andreasen et al. 1991) | DOI: 10.3109/03009749109096802 | Not available |
| Gendreau 2005 (Gendreau, Thorn et al. 2005) | PMID: 16206355 | Not available |
| Gilron 2016 (Gilron, Chaparro et al. 2016) | DOI: 10.1097/j.pain.0000000000000558 | Not available |
| Giordano 1999 (Giordano, Geraci et al. 1999) | DOI: 10.1016/S0011-393X(99)90008-5 | Not available |
| Goldenberg 1986 (Goldenberg, Felson et al. 1986) | DOI: 10.1002/art.1780291110 | Not available |
| Goldenberg 1996 (Goldenberg, Mayskiy et al. 1996) | DOI: 10.1002/art.1780391111 | Not available |
| Hannonen 1998 (Hannonen, Malminiemi et al. 1998) | DOI: 10.1093/rheumatology/37.12.1279 | Not available |
| Kempenaers 1994 (Kempenaers, Simenon et al. 1994) | DOI: 10.1159/000119138 | Not available |
| Mease 2008 (Mease, Russell et al. 2008) | PMID: 18278830 | Not available |
| Mease 2009 (Mease, Clauw et al. 2009) | doi:10.3899/jrheum.080734) | Not available |
| Moldofsky 2010 (Moldofsky, Inhaber et al. 2010) | doi:10.3899/jrheum.091041 | Not available |
| Moldofsky 2011 (Moldofsky, Harris et al. 2011) | doi:10.3899/jrheum.110194 | Not available |
| Nørregaard 1995 (Nørregaard, Volkmann et al. 1995) | DOI: 10.1016/0304-3959(94)00218-4 | Not available |
| Ohta 2012 (Ohta, Oka et al. 2012) | PMID: 23062189 | NCT00830167 |
| Olin 1998 (Olin, Klein et al. 1998) | DOI: 10.1007/BF01452251 | Not available |
| Pauer 2011 (Pauer, Winkelmann et al. 2011) | doi:10.3899/jrheum.110569 | Not available |
| Potvin 2012 (Potvin, Morin et al. 2012) | DOI: 10.1097/JCP.0b013e318267b8ca | Not available |
| Rossini 2007 (Rossini, Di Munno et al. 2007) | PMID: 17543140 | Not available |
| Roth 2012 (Roth, Lankford et al. 2012) | DOI 10.1002/acr.21595 | Not available |
| Russell 2009 (Russell, Perkins et al. 2009) | DOI 10.1002/art.24142 | Not available |
| Russell 2011 (Russell, Holman et al. 2011) | DOI: 10.1016/j.pain.2010.12.022 | Not available |
| Spaeth 2012 (Spaeth, Bennett et al. 2012) | DOI:10.1136/annrheumdis-2011-200418 | Not available |
| Vaerøy 1989 (Vaerøy, Abrahamsen et al. 1989) | DOI: 10.1007/BF02030081 | Not available |
| Vitton 2004 (Vitton, Gendreau et al. 2004) | DOI: 10.1002/hup.622 | Not available |
| Wolfe 1994 (Wolfe, Cathey et al. 1994) | DOI: 10.3109/03009749409103725 | Not available |
| Yeephu 2013 (Yeephu, Suthisisang et al. 2013) | doi: 10.1345/aph.1R725 | Not available |
| Younger 2013 (Younger, Noor et al. 2013) | DOI 10.1002/art.37734 | Not available |
| Castel et al 2012 (Castel, Cascón et al. 2012) | DOI 10.1007/s00296-016-3473-8 | Not available |
| Edinger et al 2005 (Edinger, Wohlgemuth et al. 2005) | DOI: 10.1001/archinte.165.21.2527 | Not available |
| Lami et al 2018 (Lami, Martínez et al. 2018) | DOI 10.1007/s10608-017-9875-4 | Not available |
| Martínez et al 2014 (Martínez, Miró et al. 2014) | DOI: 10.1007/s10865-013-9520-y | Not available |
| McCrae et al 2019 (McCrae, Williams et al. 2019) | PMID: 30496533 | NCT02001077 |
| Miró et al 2011 (Miró, Lupiáñez et al. 2011) | DOI: 10.1177/1359105310390544 | Not available |
| Sanchez et al 2012 (Sánchez, Díaz-Piedra et al. 2012) | Int J Clin Health Psychol, Vol. 12. Nº 1 (no NCT/PMID/DOI) | Not available |
| Saral et al 2016 (Saral, Sindel et al. 2016) | DOI: 10.1007/s00296-016-3473-8 | Not available |

Table S1. Study identification number and clinical trial registration. (PMID = PubMed ID, DOI = digital object identifier, NCT = National clinical trial registration number**)**


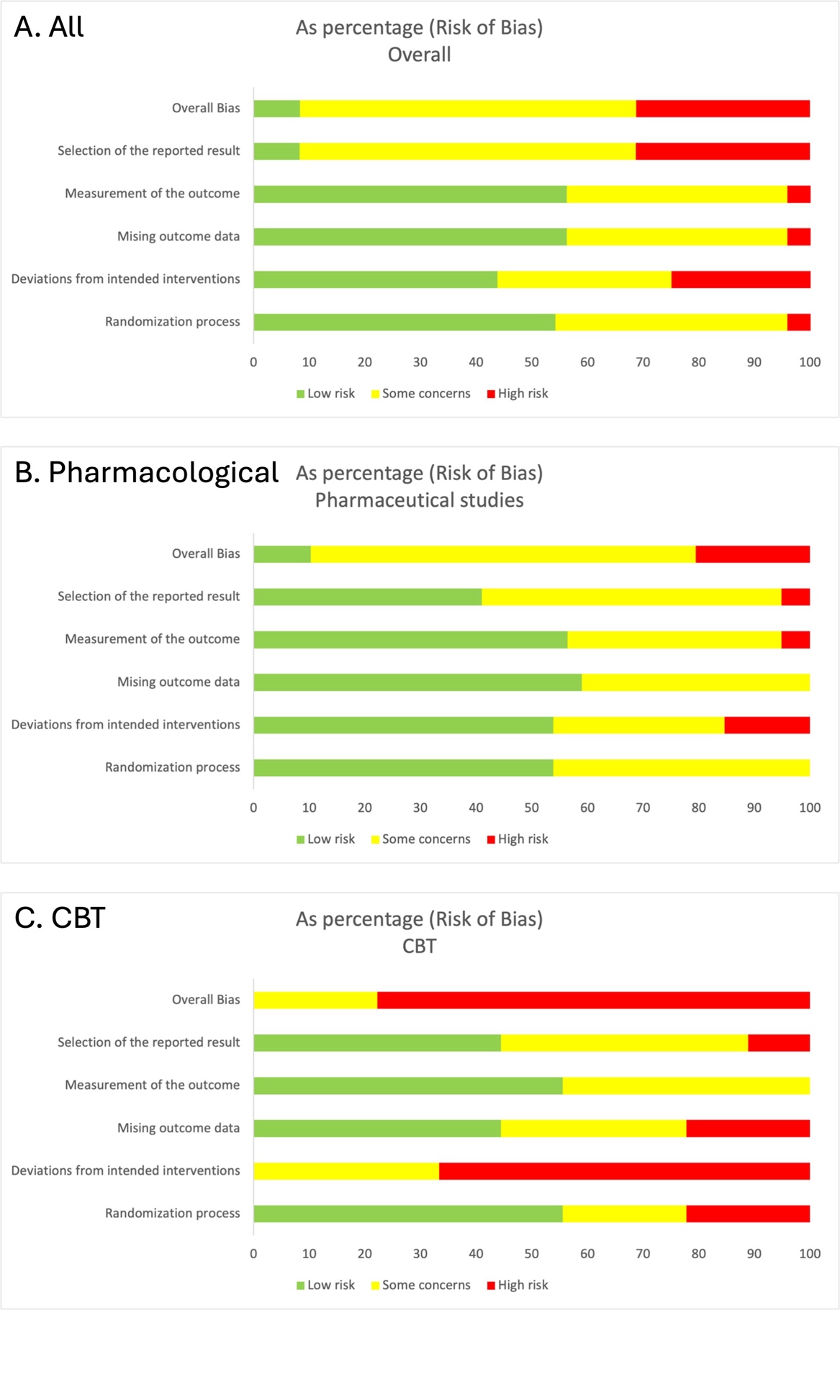


Figure S1 **Risk of Bias for Included Studies.**

 This figure provides a comprehensive summary of risk of bias across all included studies (A), and stratified for pharmacological (B) and cognitive behavioural therapy (CBT) studies (C).

*
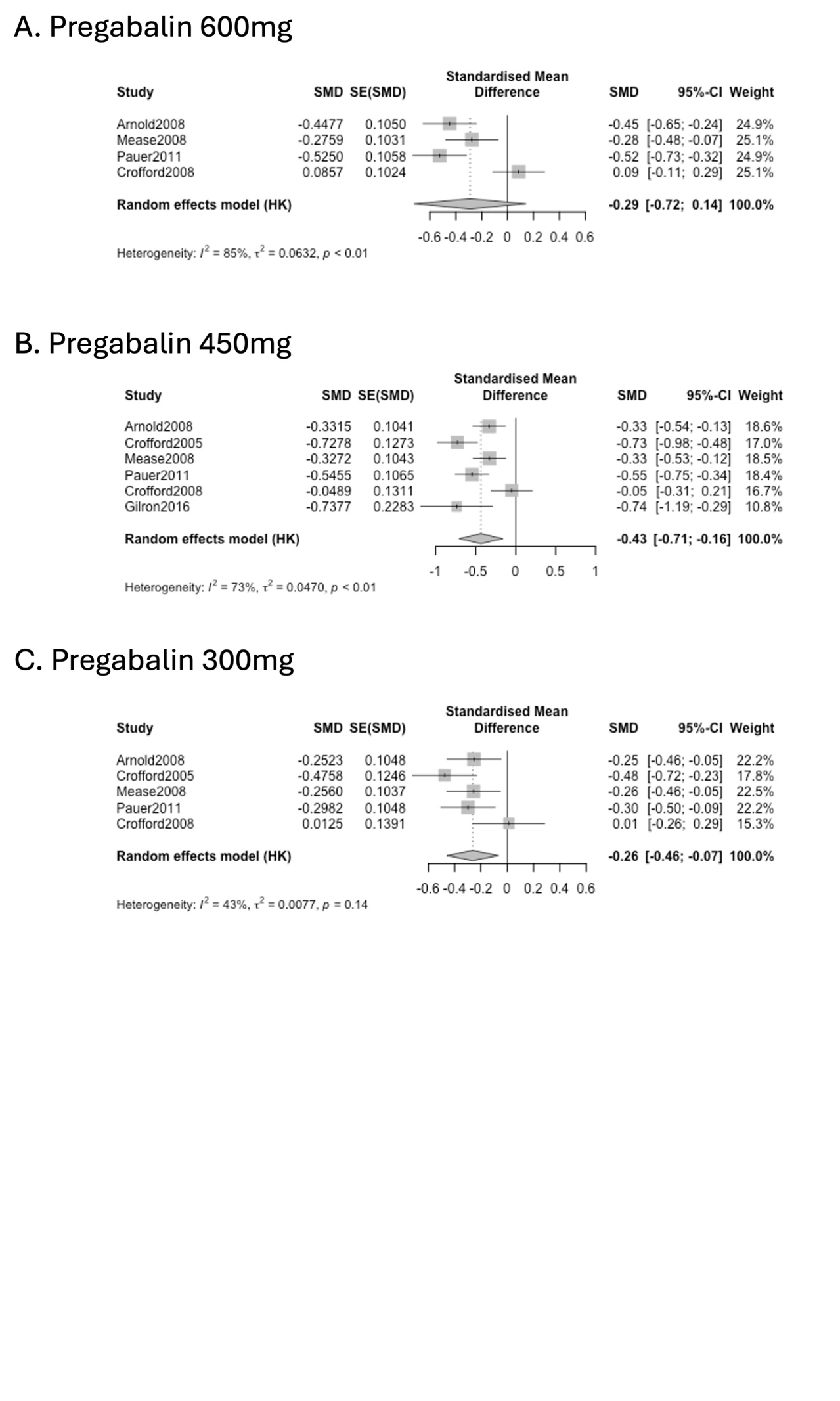
*

*Figure S2. Forest plot showing the sensitivity analysis for studies of different pregabalin dosages in fibromyalgia treatment.*

*The sensitivity analyses of pregabalin's effects on fibromyalgia symptoms reveal consistent findings across different dosage levels and study types, highlighting variations in effectiveness depending on the criteria applied. For instance, higher dosages such as 600 mg (A) and 450 mg (B) generally show more pronounced effects compared to lower doses like 300 mg (C), aligning with an expected dose-response relationship. Across all analyses, moderate-to-high heterogeneity (I² values ranging from 43% to 85%) suggests variability among studies, likely due to differences in patient populations, study designs, and outcome measures.*


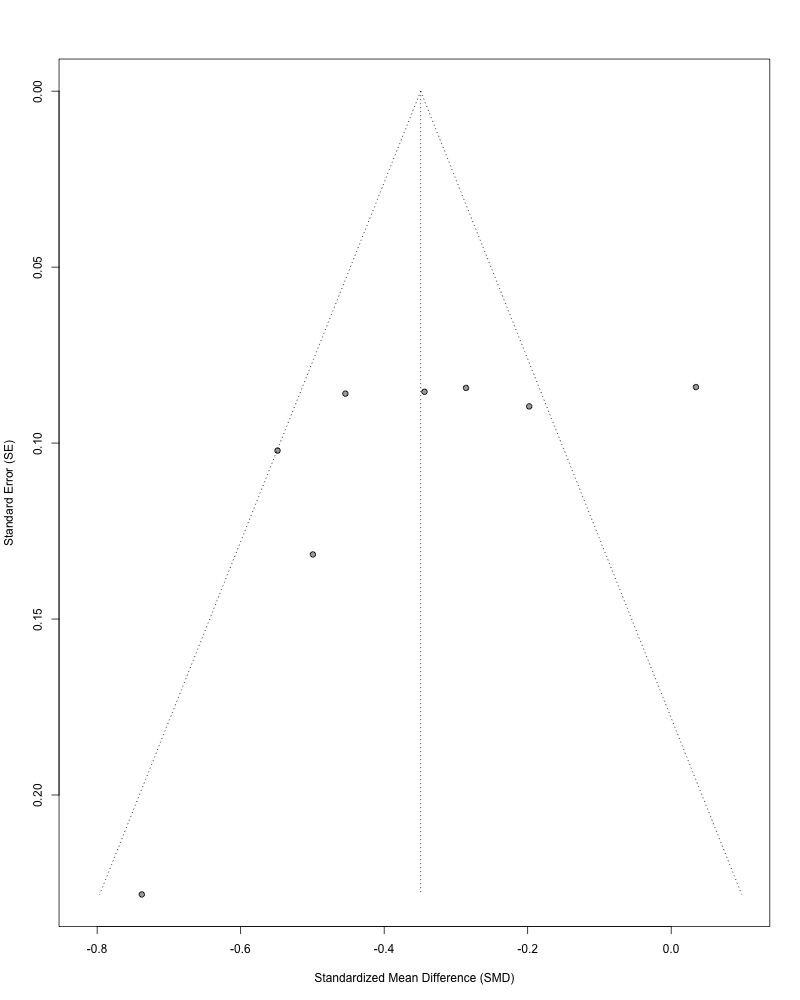


Figure S3. Funnel plot assessing publication bias for pooled studies examining pregabalin's effect on fibromyalgia symptoms.

The plot displays standardised mean differences (SMD) against standard errors (SE). Symmetry around the vertical line, representing the pooled effect size, suggests minimal publication bias. However, slight asymmetry at the lower left indicates potential for small-study effects or selective reporting, as smaller studies with larger effects are more prominent on the left side of the plot. Further investigation may be needed to confirm any bias.

*
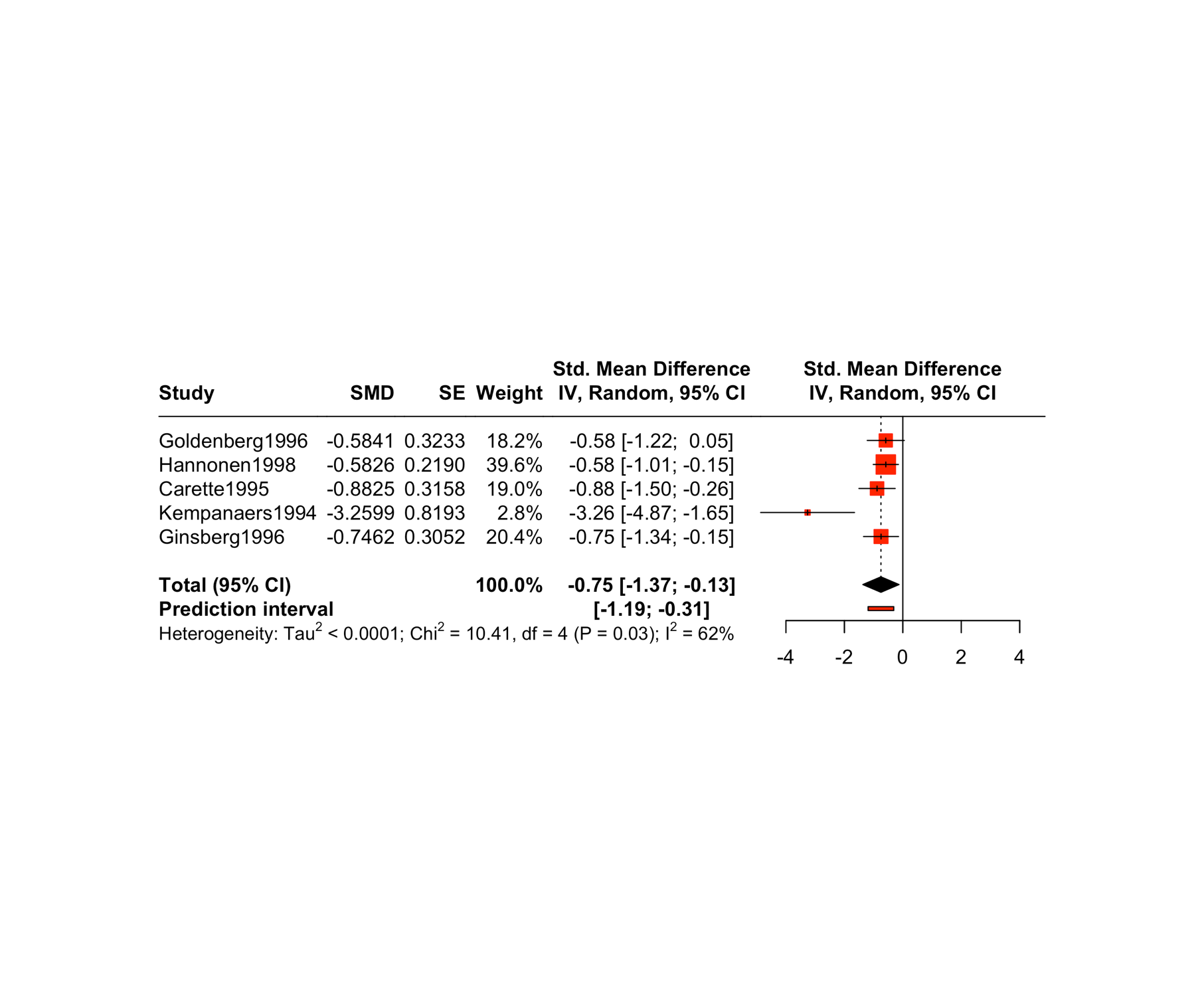
*

*Figure S4. Forest plot showing the sensitivity analysis omitting study by Braz et al. (2013).*

*A sensitivity analysis was conducted to assess the influence of individual studies on the pooled effect estimate. Influence analysis identified Braz et al. (2013) as a highly influential study, contributing disproportionately to heterogeneity. Excluding this study resulted in a stronger negative effect estimate and reduced heterogeneity, suggesting a more consistent treatment effect across remaining studies.*

*The diamond at the bottom represents the overall pooled effect, with its width corresponding to the 95% confidence interval (CI). The size of each square reflects the weight of each study in the meta-analysis. Standardised mean differences (SMDs) were calculated using baseline-adjusted SMDs to account for pre-treatment differences between intervention and control groups. A random-effects model with Hartung-Knapp (HK) adjustment was applied to ensure robust estimation of uncertainty.*


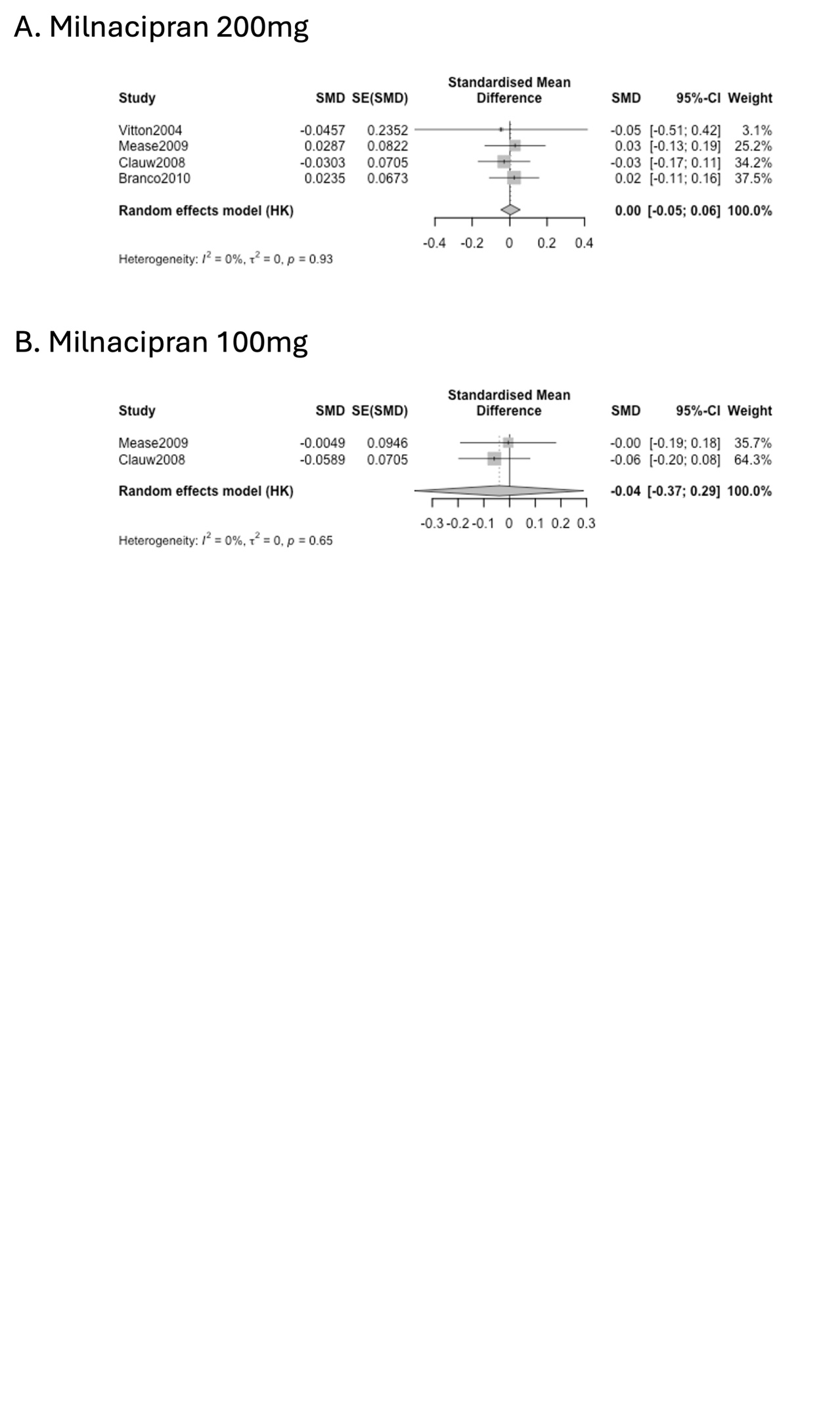


*Figure S5. Forest plot showing the sensitivity analysis for studies of different milnacipran dosages in fibromyalgia treatment.*

The sensitivity analyses for milnacipran, examining different dosages and study designs, showed small and statistically non-significant effects on sleep outcomes for fibromyalgia patients. When separated by dosage, both 200 mg (A) and 100 mg (B) groups revealed minor effects, with overlapping confidence intervals indicating no clear advantage of a higher dose. Additionally, heterogeneity across studies was low, as reflected by I² values close to 0%, suggesting consistency in findings across the milnacipran studies included in the analysis. However, the pooled analysis still demonstrated minimal overall effect.

###


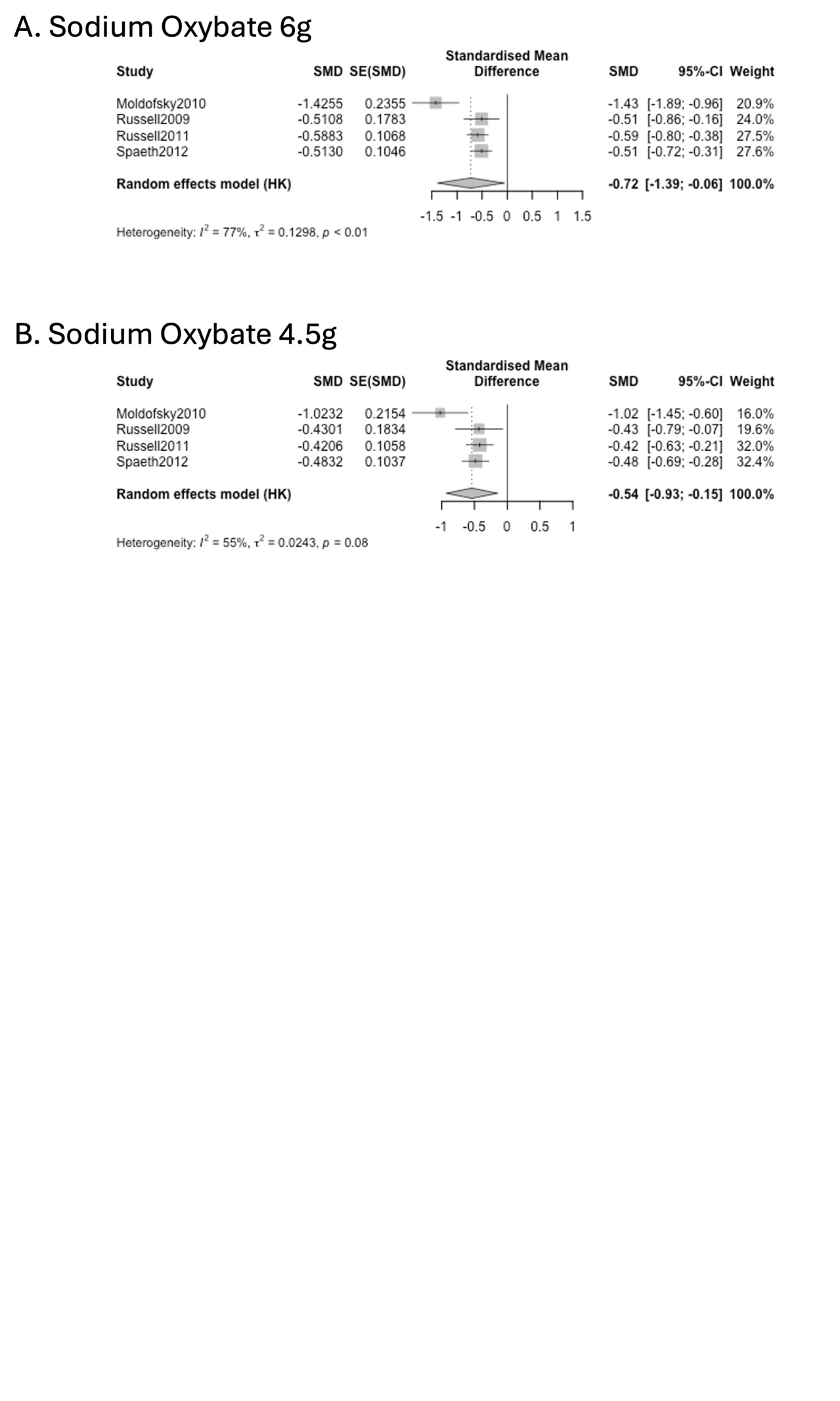


*Figure S6. Forest plot showing the sensitivity analysis for studies of different sodium oxybate dosages in fibromyalgia treatment.*

*The sensitivity analyses for sodium oxybate indicate that both 6.0 g (A) and 4.5 g (B) dosages have significant positive effects on sleep outcomes in fibromyalgia. The 6.0 g dose shows a larger effect size (SMD=-0.72), though with high heterogeneity (I^2^=77%), while the 4.5 g dose has a moderate effect (SMD=-0.54) with moderate heterogeneity (I^2^=55%).*


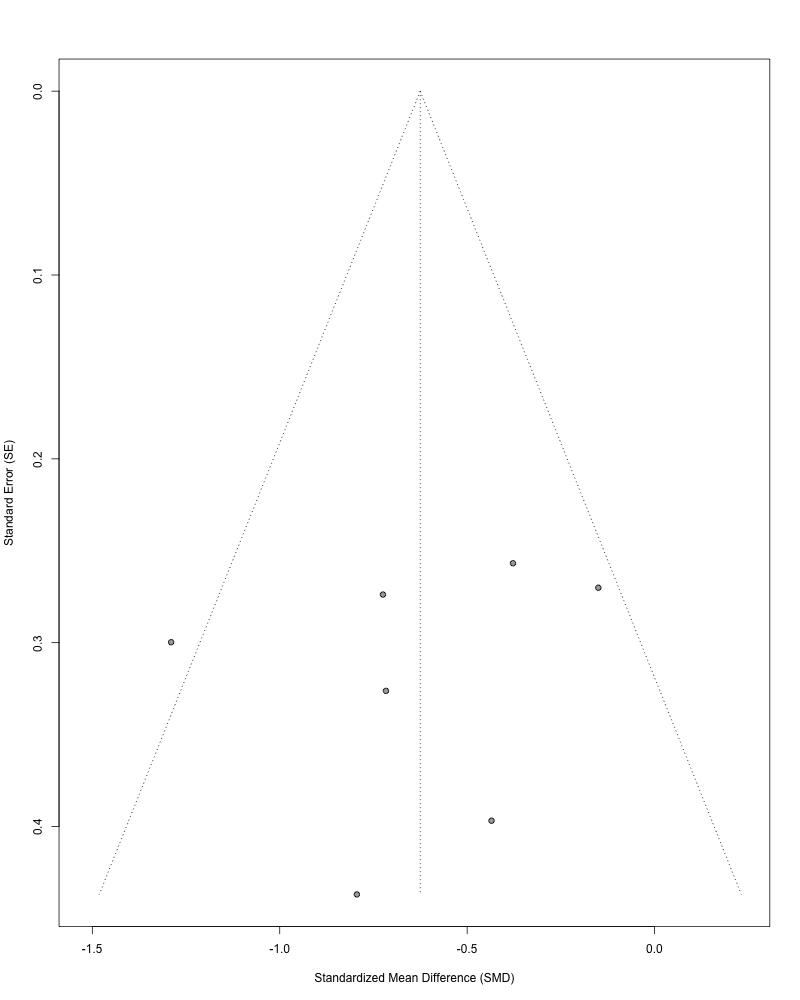


Figure S7. Funnel plot for the pooled analysis of Cognitive Behavioural Therapy for insomnia (CBT-I) interventions.

Each point corresponds to a study's standardised mean difference (SMD) and standard error (SE). The plot’s symmetry suggests minimal publication bias, reinforcing the reliability of the pooled CBT-I effect size.
